# A clinical neuroimaging platform for rapid, automated lesion detection and personalized post-stroke outcome prediction

**DOI:** 10.1038/s41746-026-02803-2

**Published:** 2026-05-27

**Authors:** Michal Brzus, Joseph Griffis, Cavan J. Riley, Joel Bruss, Carrie Shea, Hans J. Johnson, Aaron D. Boes

**Affiliations:** 1 Department of Electrical and Computer Engineering, The University of Iowa, Iowa City, IA USA; 2 Department of Pediatrics, Carver College of Medicine, The University of Iowa, Iowa City, IA USA; 3 Department of Neurology, Carver College of Medicine, The University of Iowa, Iowa City, IA USA; 4 Department of Biomedical Engineering, The University of Iowa, Iowa City, IA USA; 5 Department of Psychiatry, Carver College of Medicine, The University of Iowa, Iowa City, IA USA; 6 Iowa Neuroscience Institute, The University of Iowa, Iowa City, IA USA

**Keywords:** Computational biology and bioinformatics, Medical research, Neurology, Neuroscience

## Abstract

Accurately predicting long-term outcomes after stroke remains a key challenge in personalized medicine. Here, we present a neuroimaging platform that forecasts individualized cognitive outcomes in patients with ischemic stroke using deep learning-based lesion segmentation and location-/network-based features. This novel, fully automated system is capable of processing raw DICOM MRI data from heterogeneous scanners and generating text-based, personalized outcome information. To demonstrate this pipeline, we trained cognitive outcome-prediction models using a large lesion cohort (*N* = 604) and applied them to an independent stroke cohort (*N* = 153). Multiple cognitive outcome predictions achieved reasonable accuracy, with 96% concordance with manual methods. A report generated by a large language model provides interpretable, patient-specific prognoses within ~3 min. This demonstrates the potential for imaging-informed prognostication to inform stroke care and guide rehabilitation strategies.

## Introduction

Stroke is a leading cause of long-term disability, with a global incidence of around 12 million new cases per year^1^. Patients with a stroke typically present with multiple deficits, which may include core cognitive functions like one’s ability to speak or understand language^2^. While there is often recovery within the first few months, it is common for some deficits to persist and contribute to long-term disabilities. In contrast, other stroke symptoms may not be apparent on initial presentation but develop in the days and weeks after a stroke, such as central post-stroke pain or post-stroke depression. Given the temporal unfolding of stroke sequelae and the high variability in recovery from early deficits, the days and weeks following a stroke can be filled with anxiety and uncertainty for patients and their loved ones.

Clinicians are currently limited in their ability to predict long-term post-stroke outcomes. Research shows that individual clinician prognostication is highly variable and often inaccurate, with significant differences not attributable to experience or subspecialty expertise^3^. Clinical tools like the NIH Stroke Scale provide some prognostic utility^4^, but they lack specificity for predicting detailed cognitive outcomes, such as memory or executive function deficits, that contribute to long-term disability^5,6^. Similarly, while predictive models have been developed for global measures of disability such as the Modified Rankin Scale and Barthel Index^7–11^, they are intended to predict global outcomes and are not able to predict impairments in specific cognitive/behavioral domains, limiting their utility for applications such as identifying personalized rehabilitation strategies.

More recently, domain-targeted prediction algorithms have been developed for post-stroke motor recovery^12,13^, but these are limited in that they require steps that are not part of routine clinical practice, and there is no automated way to incorporate lesion anatomy into the prognostication. Moreover, these algorithms only address motor outcomes and do not provide a generalizable framework that can be applied to cognition and other outcomes that significantly influence quality of life and disability. Despite widespread recognition of the need for evidence-based clinical prognostication tools^14^, clinicians currently lack access to automated tools that can aid them in making personalized, post-stroke predictions of functional outcomes in multiple cognitive and behavioral domains.

Developing a clinically viable tool for automated outcome prognostication could help patients and clinicians navigate post-stroke life more effectively. An automated tool to inform outcome prognostication could help guide management decisions in the inpatient setting, inform rehabilitation interventions, and help plan more effectively for the level of services that may be required. Accurate prognostic information could also inform personalized anticipatory guidance, such as education and increased surveillance of post-stroke pain or mood symptoms for individuals at higher risk for these symptoms. Finally, providing outcome information to patients shortly after having a stroke could motivate earlier initiation of rehabilitation when patients may be most likely to respond.

It is critical to account for the stroke anatomy to enable accurate prognostication. For instance, a lesion that involves the corticospinal tract is strongly associated with poorer recovery of motor function^15,16^. In contrast, lesions that spare this tract tend to have a much better motor prognosis. The same principle applies to brain structures and networks serving other functions. Work by our group and by others has identified many critical anatomical regions and distributed networks associated with different types of impairments^2,17–30^. Importantly, statistical models can be trained to leverage lesion location, lesion-associated networks, and other non-lesion factors (e.g. demographic information) to generate personalized outcome predictions^30–33^. Recent work has also used ensemble modeling approaches to combine deep learning-based imaging models with machine learning models that leverage clinical and demographic data to predict 90-day global functional outcomes^10^, highlighting the potential of combining imaging and non-imaging variables using advanced modeling approaches to improve post-stroke outcome-prediction. However, despite these advances in lesion research for prognostication, there are still barriers to direct clinical implementation. For example, existing approaches require specialized image processing steps (e.g. lesion segmentation, image registration, intensity normalization) and/or manual interventions that create obstacles for direct clinical use. As a result, the well-established corpus of knowledge about lesion-behavior relationships has not been successfully translated into practical clinical applications.

To address this gap, we present a framework for an automated clinical tool that can leverage raw clinical imaging to generate personalized and anatomically informed outcome predictions in acute ischemic stroke, building upon our previous work^18,32,34^ and recent work by other groups^10^. Starting with raw MRI data routinely acquired for stroke management, our platform automatically identifies and segments the stroke lesion and uses the lesion location, derived network features, and patient demographic information as inputs to pre-trained machine learning models that generate predictions about long-term neuropsychological outcomes. A personalized report is automatically generated for each individual that describes the anatomical structures affected by the lesion location, its arterial distribution, and displays an image of the stroke and lesion segmentation for clinician verification.

As a proof of concept, we trained brain-behavior models to predict impairment status on an array of neuropsychological outcome measures spanning different cognitive domains in a large cohort of adult patients with chronic brain lesions (*N* = 604), embedded them into the platform, and applied the complete end-to-end prototype tool to acute clinical imaging data from an independent test cohort of patients with ischemic stroke (*N* = 153), obtaining personalized outcome predictions for each patient in the independent cohort. We demonstrate robust performance of the processing pipeline across diverse clinical scanners and acquisition sequences. We also show that our anatomically informed modeling approach can achieve meaningful and statistically significant outcome-prediction performance when applied to independent clinical imaging datasets, and that incorporating additional information about affected networks and patient demographics can improve prediction performance beyond that afforded by lesion location alone. Altogether, this work provides strong proof of concept for our approach and represents an important step toward implementing tools for automated, imaging-guided personalized outcome prediction in acute stroke care.

## Results

**Fig. 1:**
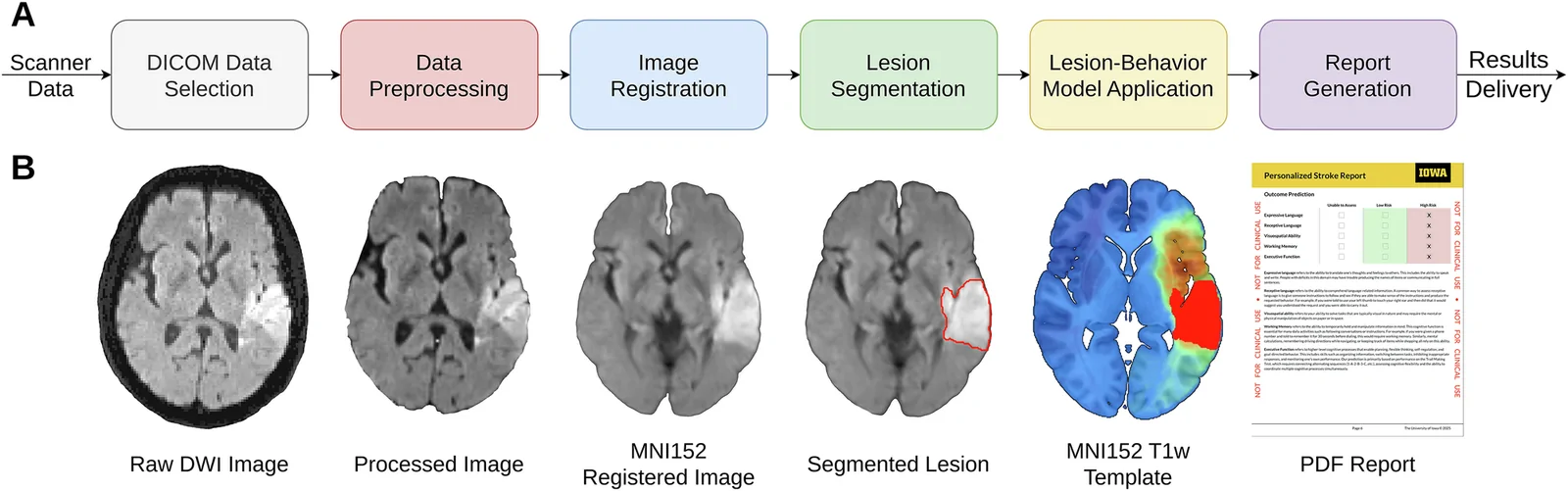
Overview of the personalized stroke outcome-prediction pipeline. **A** Diagram illustrating the end-to-end data flow, from raw scanner data to the final prediction report, highlighting key system components. **B** The diffusion-weighted imaging sequence is shown at various processing stages, including the unprocessed image, processed image, MNI152-registered image, segmented lesion, MNI152 T1w template with lesion-symptom map and binary lesion mask, and the final PDF report. Detailed descriptions of each step are provided in the text and *Supplementary Material*.

To create a clinically viable system for personalized stroke outcome prediction, we developed a fully automated pipeline that processes raw clinical MRI scans (DICOM format), segments the stroke lesion, predicts impairment status on clinical outcome measures, and generates comprehensive outcome reports (Fig. 1).

Results are divided into separate sections describing the performance of the data processing and lesion segmentation pipeline and the performance of the cognitive outcome-prediction models. In each case, the performance was evaluated on the test cohort of 160 patients with new-onset acute ischemic stroke with clinically acquired MRI scans, 153 of whom had data for any cognitive outcome measure. We report results from a single, fully automated processing run on the out-of-sample clinical test dataset.

### Data preprocessing

To prepare clinical MRI scans for stroke outcome prediction, we automatically selected relevant imaging modalities. The system requires at least one structural scan (T1-weighted, T2-weighted, or FLAIR) and diffusion-weighted imaging (DWI) with apparent diffusion coefficient (ADC) maps. Our pipeline ensures data quality by rejecting inadequate scans, uses deep learning to isolate brain tissue, and aligns images to a standard brain template (MNI-152) for anatomical analysis. These automated steps, detailed in the Methods section, enable robust data preparation for lesion segmentation and subsequent anatomically informed outcome prediction.

All 160 ischemic stroke cases in the test dataset met minimum data eligibility criteria. The pipeline successfully identified all DICOM sequences using an automated classification framework, transformed them into NIFTI format, and applied deep learning algorithms to compute brain masks and normalized images. Using quality assurance metrics from the MIQA tool and custom cross-validation rules, we successfully selected high-quality images for each subject, excluding those with artifacts or insufficient brain coverage. This process resulted in 100% of test cases being successfully registered to the MNI-152 template, leveraging high-quality synthesized T1w images to enhance registration accuracy. Intermediate outputs, including image classifications, brain masks, and registrations, were visually reviewed to confirm quality across all cases.

### Lesion segmentation

**Fig. 2:**
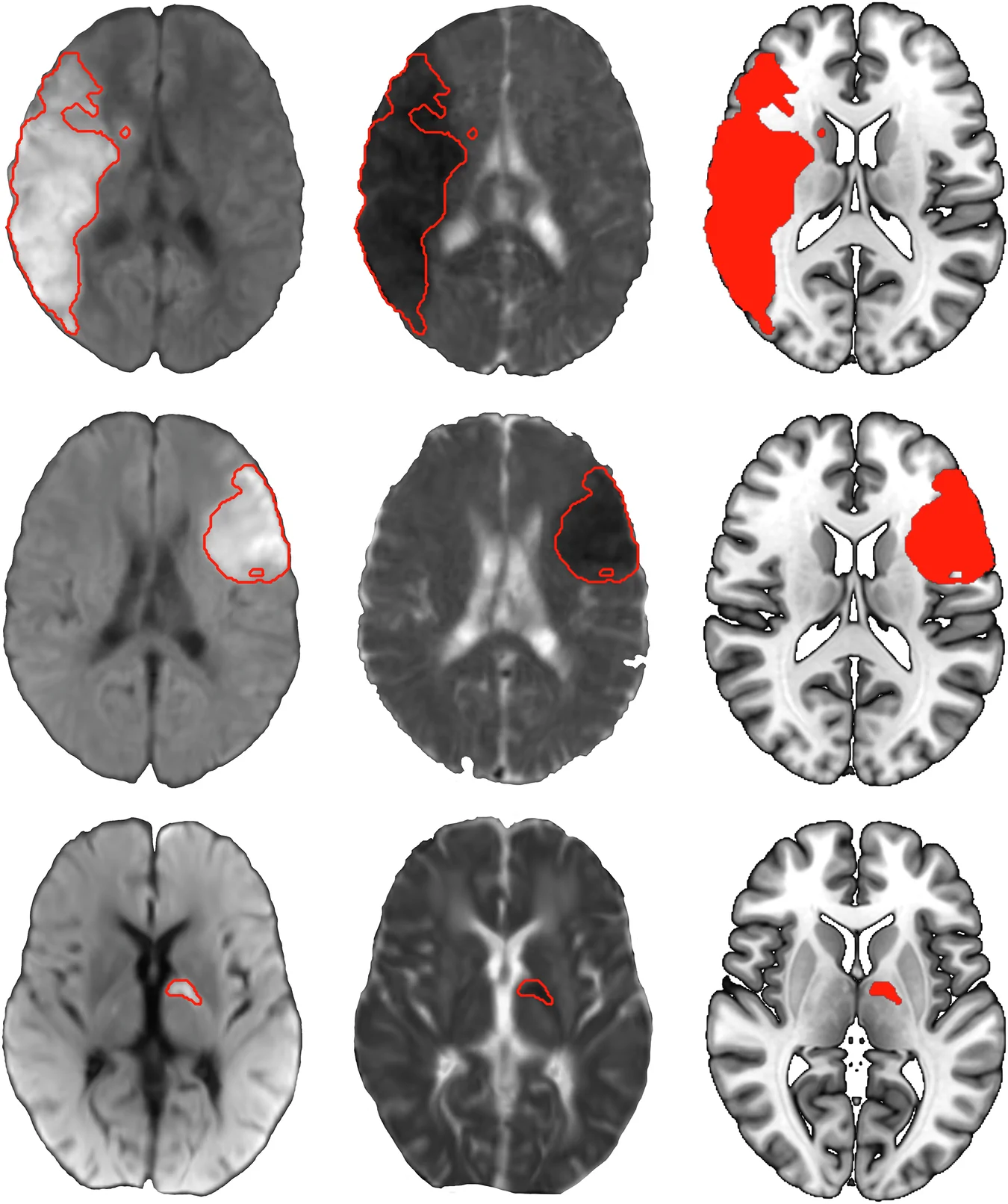
Examples of automated lesion segmentations across three different cases. Axial slices of diffusion-weighted imaging (left column) and apparent diffusion coefficient (middle column) images in MNI152 space are input to a deep learning model to produce binary lesion masks, outlined in red. The corresponding MNI152 T1w template images (right column) demonstrate the segmented lesions.

Automated lesion segmentation was enabled by training a 3D UNet deep learning model with residual connections on 700 subjects using DWI and ADC images with manually labeled binary lesions. Example lesion segmentations on test subjects are shown in Fig. 2. Lesion segmentation performance was evaluated in three ways using a subset of patients with manually traced lesions (*N* = 57 of 160). First, we evaluated the model’s performance identifying acute infarcts, which revealed detection of 93% of lesions ≥ 1 cm³ and 98% of lesions ≥ 2.5 cm³. Second, segmentation quality was evaluated with a Dice coefficient, achieving a mean of 0.69 (SD = 0.21). For imaging data acquired post-2015, the mean Dice score improved to 0.74 (Supplementary Tables 4–5). Third, to confirm that model-generated segmentations maintain predictive capability, we compared prognostic classification similarity between outcome-prediction models using automated versus manual segmentations, yielding a 96% average correspondence across 681 individual cognitive outcome predictions (Supplementary Fig. 6). This indicates that automated segmentations consistently capture behaviorally relevant anatomical features comparable to manual segmentations.

### Cognitive outcome predictions

**Fig. 3:**
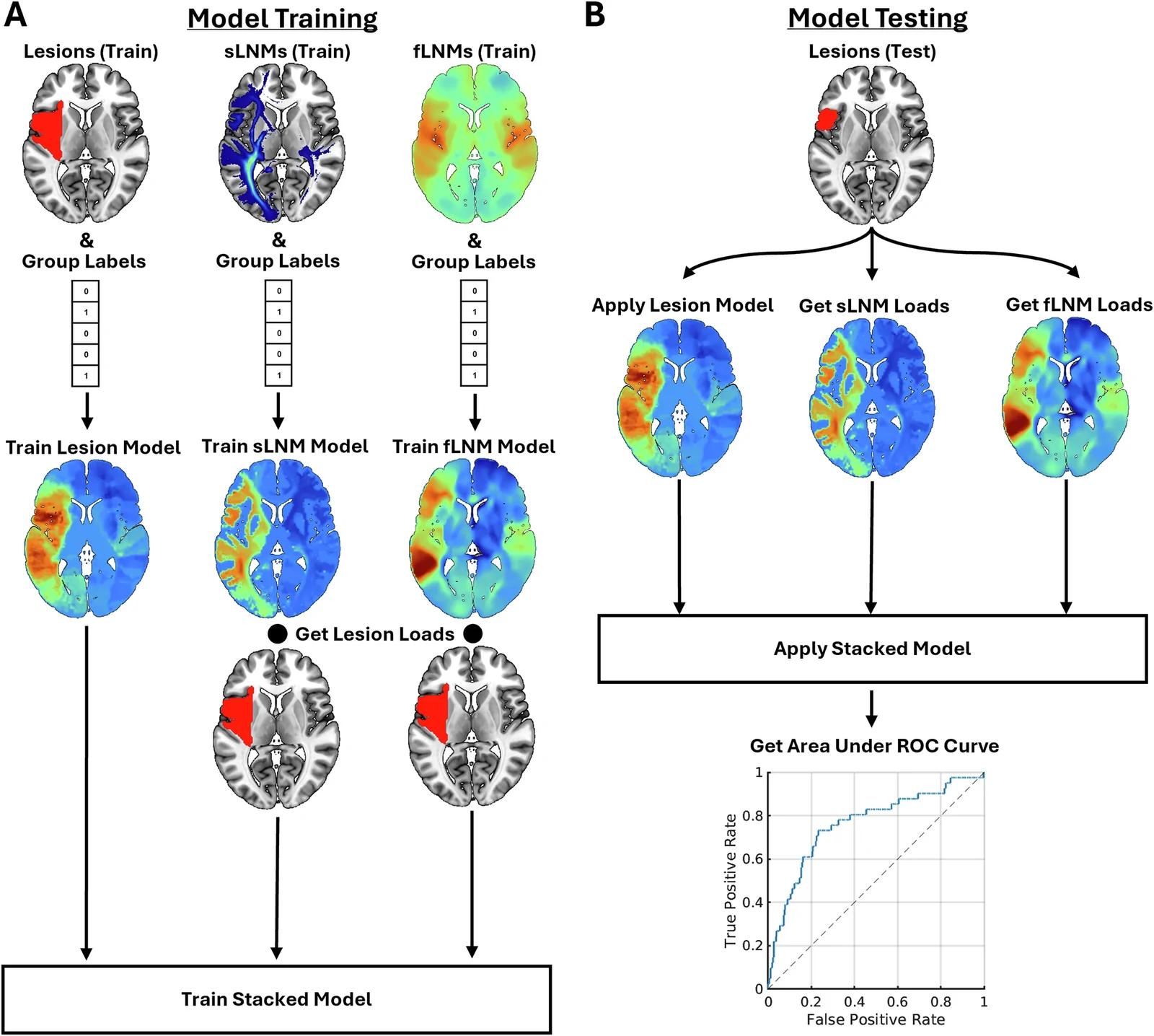
Cognitive outcome-prediction modeling methods. **A** Model training procedure. Partial least squares classification models were trained to classify patients as either ‘impaired’ or ‘unimpaired’ using lesion location and derived structural lesion-network map (sLNM) or functional lesion-network map (fLNM) features. A separate model was trained for each feature. For network models, “lesion load” features were computed as the dot product between the vectorized patient lesion segmentations and vectorized model coefficient maps. Finally, stacked ridge-regularized logistic regression models were trained using the predicted group labels from the lesion models and lesion loads on the sLNM and fLNM models as predictor features, combining information about lesion location and lesion-derived networks. **B** Model testing procedure. Models were evaluated by applying the trained models to lesion masks and ‘lesion load’ features (computed on the coefficient maps from the models generated using the training dataset) from the test dataset, obtaining predicted classification scores and predicted group labels for each patient in the test dataset. The predicted classification scores were then used along with the true labels to construct a Receiver Operating Characteristic (ROC) curve, and model performance was evaluated by computing the area under the ROC curve.

We trained multivariate classification models to predict impairment status on 28 different neuropsychological outcomes using lesion location, lesion-derived networks, and demographic information from 604 patients with chronic brain lesions from the Iowa Lesion Registry. We applied these models to predict chronic ( > 3 month) impairment status in a sample of 153 acute ischemic stroke patients with lesion segmentations generated by our automated pipeline. Training and test set sample sizes varied across outcome measures and are reported in Supplementary Table 7. The approach to model training and testing is summarized in Fig. 3.

**Fig. 4:**
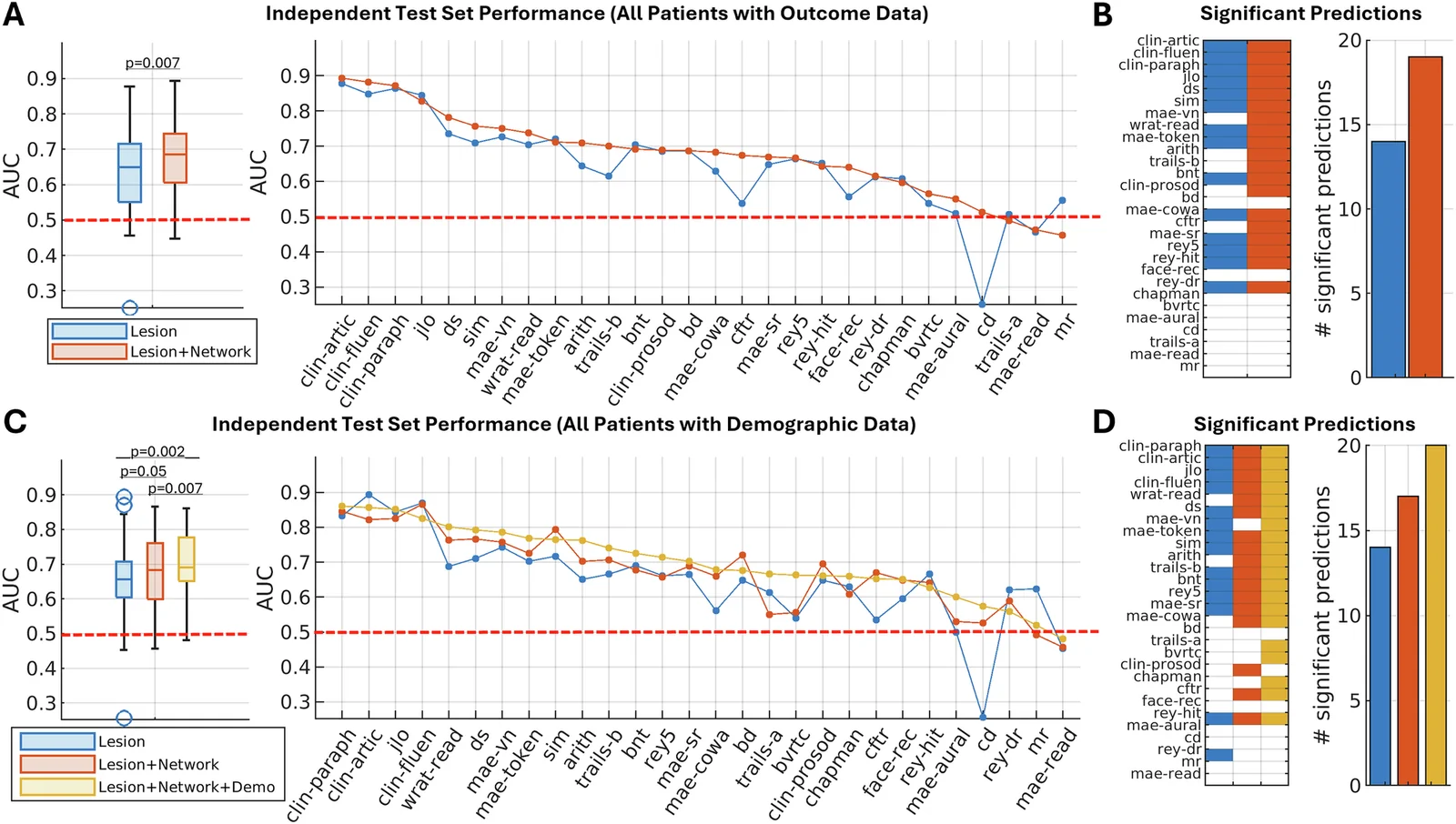
Cognitive outcome-prediction results. **A**. Test dataset AUCs for the lesion location and stacked lesion + network models. The boxplots in the left panel show the distributions of AUCs across all outcome measures for each modeling strategy. The line plots in the right panel show AUCs for each outcome measure, sorted in descending order based on performance of the stacked lesion + network models. *P* values reflect results of Wilcoxon signed-rank tests comparing AUC distributions across tasks (*N* = 28). The dashed red lines correspond to chance performance levels that would be obtained by random guessing. **B**. The colored cells in the matrix correspond to statistically significant (*p* < 0.05) test set outcome predictions as determined by permutation testing, while white cells indicate statistically non-significant (*p* > 0.05) test set outcome predictions. The bar graph shows the total number of statistically significant outcome predictions for each modeling strategy. **C**, **D**. Same as (**A**, **B**), but for analyses evaluating whether adding demographic information (age, education) to the stacked lesion + network models can further increase performance. These analyses were restricted to the subset of patients with complete demographic data (36 training set patients excluded, 10 test set patients excluded). Full names of each outcome are provided in the *Supplementary Material*. Note—for panels (**B**, **D**), the outcomes on the y-axes are ordered the same way as those on the x-axes in panels (**A**, **C**).

We evaluated the classification performance for each neuropsychological outcome by applying the trained model to the test dataset, constructing a receiver-operator characteristic (ROC) curve using the predicted classification scores and true class labels, and then computing the area under the curve (AUC). AUC scores of 0.5 correspond to the performance that would be expected from a random classifier, and higher values reflect more accurate classification performance. Outcome-prediction results from the independent test dataset are summarized in Fig. 4, and results obtained from cross-validation analyses in the training dataset are shown in Supplemental Fig. 5. AUCs varied substantially across the 28 outcomes evaluated in the training dataset. Many models achieved statistically significant (*p* < 0.05) classification performance as determined by permutation testing, with select models achieving excellent performance (i.e. AUCs ≥ 0.8), many models achieving intermediate performance (i.e. 0.6 < AUC < 0.8), and a few models performing worse than would be expected from a random classifier (i.e. AUCs < 0.5).

We tested whether the inclusion of lesion-derived network features and demographic information could improve performance beyond that afforded by lesion location alone. In line with prior work^10^, models that incorporated both lesion location and lesion network information often performed better than models that relied on lesion location alone (Fig. 4A, B). Across outcomes, models incorporating lesion and network information achieved significantly higher AUC values than models relying on lesion location alone (signed-rank test, *N* = 28 outcomes, signed rank = 86, *p* = 0.007). The lesion + network models also achieved more statistically significant (i.e., permutation *p* < 0.05) classification results in the test dataset than the lesion-only models (Fig. 4C), indicating that the addition of network information enabled more robust classification performance. Adding demographic information (age, education) further improved performance (Fig. 4C, D). Models incorporating lesion, network, and demographic information achieved significantly higher AUC values across outcomes than both the lesion + network models (signed-rank test, *N* = 28 outcomes, signed rank = 86, *p* = 0.007) and the lesion-only models (signed-rank test, *N* = 28 outcomes, signed rank = 69, *p* = 0.002), and achieved more statistically significant (permutation *p* < 0.05) outcome predictions (Fig. 4D). These results demonstrate that incorporating lesion-derived network information and demographic data can enhance cognitive outcome-prediction beyond what is afforded by lesion location alone.

**Fig. 5:**
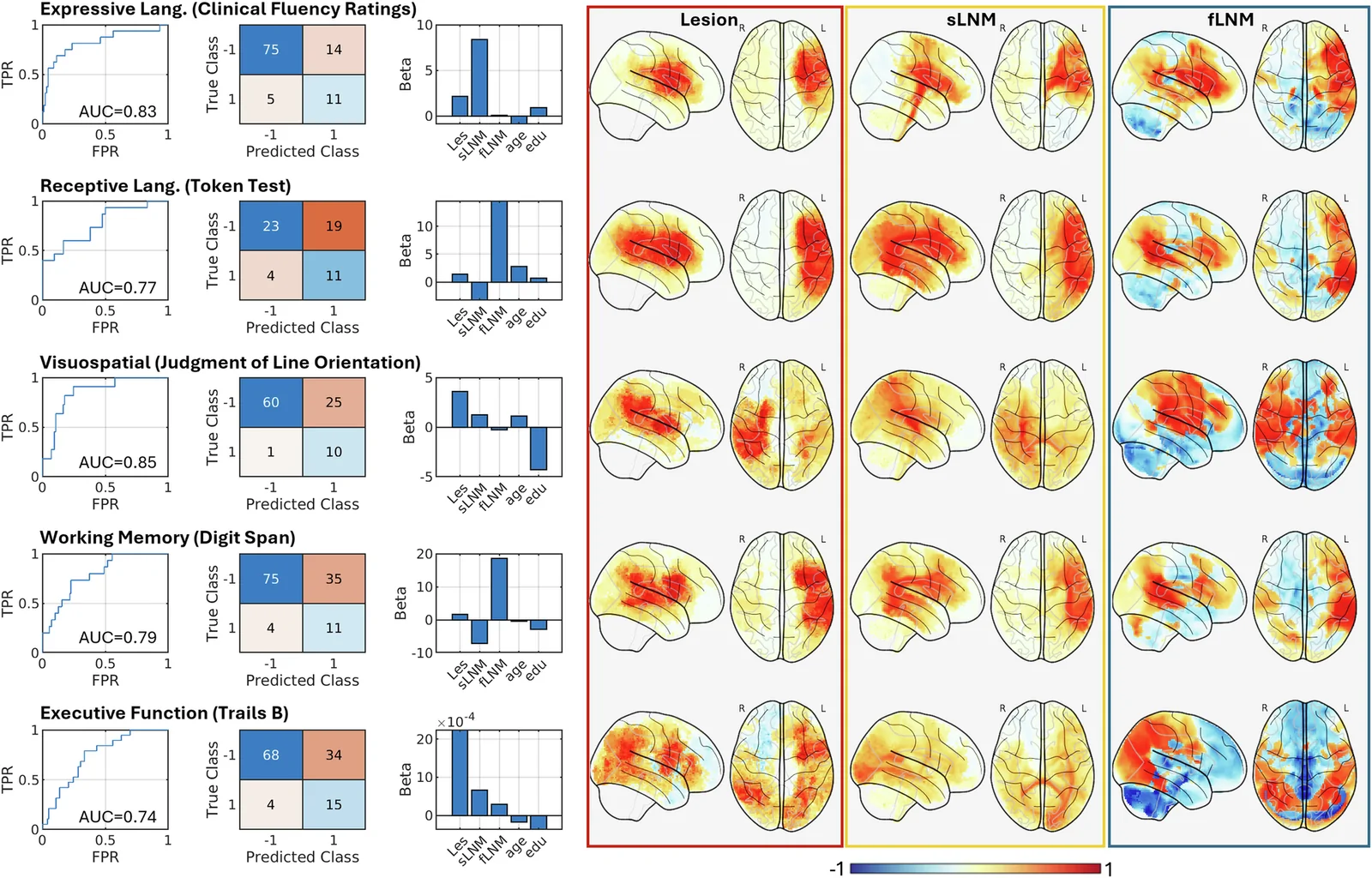
Detailed breakdown of 5 selected cognitive outcomes. Each row corresponds to a different outcome. The line plots show the ROC curve for each outcome, obtained by applying the trained model to the test dataset. The matrices show classification performance in the test dataset using the optimized classification score thresholds from the training dataset. The positive class corresponds to impaired patients, and the negative class corresponds to unimpaired patients. The bar plots show the model coefficients from the stacked logistic ridge regression models. Surface plots show the full (unthresholded) voxel-wise partial least squares classification model coefficients from the individual lesion location, functional lesion network map (fLNM), and structural lesion network map (sLNM) models. Partial least squares classification model weights were normalized to the range [−1, 1] by dividing all weights in each map by the maximum absolute weight value for visualization purposes.

Figure 5 shows detailed characterizations for 5 models targeting distinct cognitive outcomes spanning expressive language (clinical fluency ratings), receptive language (Token Test), visuospatial (Judgment of Line Orientation), auditory working memory (Digit Span), and executive function (Trails B) domains. In general, each of these models performed well in correctly classifying impaired patients in the test dataset (i.e., sensitivity) at the optimized classification score thresholds identified in the training dataset, but they exhibited more variable performance for correctly classifying unimpaired patients (i.e., specificity). Some models, such as the model for Judgment of Line Orientation, achieved good performance for both groups. This model correctly classified 91% of impaired patients and 71% of unimpaired patients, demonstrating good sensitivity and specificity. Other models, however, demonstrated good sensitivity but poor specificity. For example, the model for the Token Test correctly classified 73% of impaired patients but only correctly classified 55% of unimpaired patients.

In the context of the moderate-to-high AUCs for these models, which indicate that the predicted classification scores can achieve robust group discrimination across classification score thresholds, these observations suggest that the optimized classification score thresholds learned from the training dataset may not always be optimal in the test dataset even if the models themselves have good predictive power. Indeed, the Token Test model exhibited much better specificity when evaluated using nested 5-fold cross-validation within the training dataset (cross-validation AUC = 0.87; specificity = 0.84; sensitivity = 0.79). Since the classification thresholds were optimized within the training dataset to minimize the average misclassification cost, the reduced specificity observed for some outcomes in the test dataset could reflect differences between the training and test datasets in terms of lesion etiology, lesion segmentation methods (automated vs. manual), and/or lesion chronicity. This could be addressed in future iterations by better matching the lesion characteristics of the training datasets with those of the test datasets and/or by explicitly calibrating the classification thresholds of the trained models in an independent calibration dataset that has lesion characteristics more closely matched to those of the test dataset. As these approaches would require the collection of additional datasets, they are beyond the scope of the current study. Nonetheless, improving the generalizability of trained classification score thresholds is an important goal for future iterations of the cognitive outcome-prediction models.

Brain-behavior associations varied substantially across the 5 models shown in Fig. 5, highlighting that the predictive value of different anatomical features varies depending on the predicted outcome. The coefficient maps from the individual models highlight variations in the predictive anatomy within domains (e.g., expressive vs. receptive language outcomes) and between domains (e.g. language vs. visuospatial). Perhaps surprisingly, they also highlight similarities between domains and suggest a partially overlapping predictive anatomy between Token Test (i.e., receptive language) and Digit Span (i.e., auditory working memory), suggesting that deficits in receptive language and deficits in auditory working memory may result from damage to the same or partially overlapping brain systems.

### Report generation

A structured PDF report summarizing key anatomical and cognitive outcome-prediction information was generated for each participant using a large language model. Each report describes the regional anatomy of the lesion, the arterial distribution, the lesion volume, and prognostic information for select cognitive domains. Multi-level DWI axial images are shown spanning the lesion and lesion segmentation to facilitate verification of the anatomy. These reports were encapsulated in DICOM format for PACS communication, with an example report shown in the *Supplementary Material*. Clinician-focused sections provide detailed neuroimaging and neuroanatomical summaries, while the final two pages are tailored for patients and families, translating complex medical findings into accessible, non-technical language using relatable analogies and avoiding medical jargon. Systematic readability analysis of the patient-directed content (SMOG index on 100 randomly selected reports from the test dataset) confirmed a mean grade level of 6.6 (SD = 0.6; range 4.9–7.8), well below the average US adult reading level and appropriate for broad patient comprehension (see Supplementary Results for full details).

All reports underwent manual technical validation to confirm correct report structure, proper inclusion of all pipeline-generated components (lesion masks, network maps, volumetric statistics, and risk predictions), and absence of hallucinations or structural mistakes. This validation verified that every report accurately reflected the underlying pipeline outputs with no discrepancies. In addition, a board-certified stroke neurologist (ADB) independently reviewed a randomly selected subset of 30 reports ( ~ 20% of the independent test cohort) from a clinical standpoint. This clinical review did not detect any reporting errors that would affect patient care or decision-making if this tool were used in a clinical context.

### Runtime analysis

End-to-end runtime performance was assessed on a workstation equipped with an Intel Xeon w7-3545 CPU (24 cores, 48 threads, 4.8 GHz max frequency) and an NVIDIA RTX 6000 Ada GPU (48GB memory). The system achieved an average end-to-end processing time of 121.3 s (approximately 2 min), with 95% of cases completing in under 3 minutes (range = 80.4–238.1 seconds, SD = 22.3), as shown in Table 1. Key pipeline stages, including data selection, registration, lesion segmentation, outcome prediction, and report generation, were optimized for efficiency, with report generation benefiting significantly from GPU acceleration of large language model inference. This rapid processing speed supports the system’s feasibility for acute stroke care settings, where timely analysis is critical. Detailed performance benchmarks, including CPU-only runtimes and individual component breakdowns, are provided in Supplementary Table 8.

**Table. 1:** Runtime summary of processing system components in seconds

System Component	Mean	Min	Max	Std
Data Selection and Preprocessing	29.6	18.8	48.0	5.0
Registration	76.7	49.1	168.8	15.4
Lesion Segmentation	4.1	3.9	4.6	0.2
Outcome Prediction	0.9	0.8	0.9	0.0
Report Generation	10.0	7.8	15.8	1.65
**Total**	121.3	80.4	238.1	22.3

## Discussion

Here, we present a comprehensive end-to-end clinical neuroimaging platform for fully automated data processing and outcome prediction in patients presenting with acute ischemic stroke. Importantly, our platform can generate personalized outcome predictions from raw clinical imaging data in less than 3 minutes and communicate results to patients and clinicians in automatically generated reports. We demonstrated robust performance of our automated processing pipeline, with all 160 MRIs from the test dataset being processed without any failures. The deep learning algorithm for stroke segmentation performed adequately, with excellent performance for lesions >1 cm^3^_._ We also demonstrated promising out-of-sample prediction performance across a range of neuropsychological outcome measures. We showed that incorporating lesion-derived network information and demographic variables can enhance prediction performance (Fig. 4) and highlighted performance for a set of outcome measures spanning distinct cognitive domains (Fig. 5). This work represents an important advance towards harnessing the translational potential of lesion-symptom research for informing stroke prognostication.

Historically, lesion-symptom mapping and related fields have primarily been concerned with identifying anatomical structures that show statistically significant group-level associations with lesion location^35,36^. However, the pursuit of personalized, precision medicine approaches to stroke prognostication requires the development of lesion-symptom models that can reliably predict outcomes in individual patients^37^. Furthermore, for such models to be clinically useful, they must be able to generalize beyond high-quality research datasets to real-world clinical data, and they must be implemented in a fully automated platform that generates outcome predictions from raw clinical imaging quickly and easily. Our approach, which embeds pre-trained brain-behavior models within a fully automated end-to-end clinical data processing pipeline, solves these issues and represents a major step towards developing imaging-based clinical tools for ischemic stroke outcome prognostication. Notably, excellent classification performance was consistently achieved for measures corresponding to clinician ratings of impairment in fluency, articulation, and paraphasias (Fig. 4, “clin-fluen”, “clin-artic”, “clin-paraph”), indicating that brain-behavior models can robustly predict chronic “real-world” clinical outcomes in a way that aligns with clinician evaluations.

Since the introduction of multivariate lesion-symptom modeling methods in the early 2010s^36,38^, a variety of models that leverage lesion location and/or other imaging-derived features such as resting-state functional connectivity or white-matter structural disconnection measures to predict diverse post-stroke outcomes have been described in the research literature^2,18,30,39–41^. We build upon this body of work and extend it by directly incorporating our pre-trained models into an end-to-end clinical tool that quickly and automatically generates individualized predictions from raw DICOM datasets, eliminating the need for specialized image processing, feature extraction, or model application by clinicians. While it is difficult to directly compare our results to those of other published models due to factors such as differences in the outcome measures, sample sizes, prediction approaches (e.g. classification vs. regression), predictive features, and validation strategies (e.g. independent validation vs. cross-validation), our results appear to be comparable to other state-of-the-art approaches reported in the literature. For example, diverse modeling strategies have yielded AUCs ranging from 0.6 to 0.9 when predicting 3-month global functional impairments based on the mRS^7,10,11^, and the best-performing teams in a recent international stroke outcome-prediction competition obtained AUCs ranging from 0.73 to 0.84 for measures of cognitive impairment (e.g. MoCA)^42^. The AUCs for the 5 domain-representative measures shown in Fig. 5 range from 0.74 to 0.85, with some individual measures achieving AUCs of 0.90 (i.e. clinical fluency ratings—Fig. 4A). This suggests that our outcome-prediction approach performs comparably to other state-of-the-art approaches reported in the literature, providing a solid foundation that we hope to continue to expand and improve upon in future iterations.

The system’s automated stroke lesion segmentation results add to a rich literature on deep learning-based approaches for acute ischemic stroke, including systematic reviews and the ISLES 2022 challenge benchmarks^43^. While direct comparisons are complicated by differences in image resolution, quality, scanner heterogeneity, in- versus out-of-sample testing, and lesion volume distributions across datasets^44^, our 3D Residual U-Net model achieved Dice scores comparable to state-of-the-art DWI/ADC-based methods (including the DeepISLES ensemble^45^, top ISLES 2022 entries, and systematic-review^46^ averages ~0.70 for heterogeneous cohorts) when evaluated on both internal datasets and a fully independent, highly heterogeneous clinical test set spanning two decades and 20 scanner models. These results support the translational robustness of our pipeline for real-world clinical deployment.

While our brain-behavior models were trained on manual lesion segmentations (and derived network features) from research datasets, they nonetheless were able to achieve meaningful and statistically significant outcome-prediction performance when applied to automatically generated lesion segmentations (and derived network features) across a variety of cognitive outcome measures, including measures of distinct cognitive domains, in a fully independent clinical test dataset (Figs. 4–5**)**. This is a strong proof-of-concept for our approach, demonstrating that lesion information derived from clinical scans can be successfully leveraged to predict chronic cognitive outcomes in individual patients. While from a machine learning perspective, it would be ideal to train brain-behavior models on acute lesion segmentations produced by the automated pipeline (i.e. to maximize the representativeness of the training dataset with respect to the data that models will be applied to in practice), most existing lesion-symptom mapping datasets consist of manual lesion segmentations from chronic research-quality structural scans. Importantly, our results suggest that models trained on manual lesion segmentations from research datasets can still produce meaningful predictions when applied to automatically generated lesion segmentations from acute clinical scans, suggesting that large existing research datasets can be leveraged to develop brain-behavior models to predict outcomes in acute ischemic stroke.

Even so, lesion-behavior relationships may vary depending on lesion chronicity and/or lesion etiology^47,48^, and it is possible that differences between the training and test datasets related to these factors could contributed to the variability observed in test set model performance. Similarly, since lesions are dynamic and change over time^47–49^, the acute lesion may not be fully representative of the lesion at the time of chronic behavioral assessment, and this could reduce the performance of models trained on chronic lesions imaged near the time of behavioral assessment. Determining whether it is better to maximize the training set sample size by including diverse lesion etiologies and/or chronicities or maximize the comparability of lesion characteristics between the training and test datasets is an important consideration for future work in this domain.

Notably, our results demonstrate that even relatively simple measures of lesion-network properties and patient demographics can yield significant and sometimes drastic improvements in prediction performance beyond what is possible with lesion location alone (Fig. 4), consistent with other recent work indicating that integrating imaging and clinical/demographic information can improve prediction of global functional outcomes after stroke^10^. We anticipate continued improvements in prediction performance as future predictive models expand the current range of both imaging and non-imaging predictors. Lesion network mapping approaches will likely continue to improve, along with the incorporation of other imaging variables with predictive utility for long-term functional outcomes, like quantifying white-matter hyperintensity^50^, large vessel occlusion^51^, cortical atrophy^52^, and brain age^53^. Tissue segmentation and normative modeling could also be leveraged to improve predictive models^54^. Similarly, deep learning methods that learn predictive features directly from anatomical scans, rather than relying on the segmented lesion alone, have the potential to further improve performance^10^. With new training datasets, it will be possible to expand the range of clinical and demographic variables included in the statistical models, including medical co-morbidities, more accurate estimation of pre-stroke baseline, social support estimates, and acute impairment measures, such as the NIH stroke scale. It may be possible for these non-imaging variables to be extracted from electronic medical records or entered manually. The ongoing collection of longitudinal outcomes from large and well-characterized stroke cohorts (e.g. DISCOVERY, VERIFY) will facilitate the development of these expanded models^55,56^.

The reports generated by the system represent a key translational advance: comprehensive manual technical validation across all cases and targeted clinical review of a 20% random sample confirmed factual accuracy, absence of hallucinations, and clinical appropriateness. This completes the end-to-end pipeline with a standardized, multimodal, PACS-compatible output that supports immediate clinical integration and consistent communication. However, we are not advocating for the clinical use of the outcome-prediction component in its current form. Instead, we view the current overall performance and proof-of-concept analysis as a promising starting point for further development. There are many limitations of the current approach that can be improved. For example, our deep learning algorithm for lesion segmentation has improved consistently as training data has been added, and the current 700 MRIs are likely not approaching peak lesion segmentation accuracy. Further, our cognitive outcome training dataset may be suboptimal as a mixed lesion etiology cohort rather than a stroke-specific cohort, especially given the very low rates of impairment for many outcomes; larger training datasets with better representation of impaired patients would be expected to improve prediction performance. Relatedly, individual cognitive/behavioral tests may not be ideal targets for eventual clinical applications. The development and refinement of models that can predict clinically relevant outcomes (e.g., expressive language, visuospatial function, motor function) instead of performance on specific neuropsychological tests is an important future direction for the pipeline that will likely require the harmonization of within-domain outcome data across different assessments and likely across multiple independent datasets.

In closing, this paper presents a novel and comprehensive automated clinical neuroimaging platform for lesion-informed prognostication of ischemic stroke. With continued development, we are optimistic that this approach can extract clinically valuable information from routinely acquired clinical neuroimaging and put it in the hands of clinicians and patients, forming a much-needed bridge connecting advances in lesion research to improvements in precision medicine.

## Methods

The fully automated clinical neuroimaging platform consists of three main integrated components. The first component is an image processing and lesion segmentation pipeline designed to operate directly on heterogeneous raw clinical MRI scans acquired during routine acute stroke care. The second component comprises statistical models that generate personalized cognitive outcome predictions based on lesion location and associated network disconnection features. The third component is an automated report generation system that integrates the imaging-derived lesion information and model predictions with large language model (LLM) technology to produce concise, clinically relevant summaries for both clinicians and patients. The minimum system requirements include one structural image (T1-weighted, T2-weighted, or FLAIR) along with diffusion-weighted imaging (DWI) and apparent diffusion coefficient (ADC) sequences. Here, we describe the complete end-to-end system and evaluate its performance on an independent clinical test cohort of 153 patients with acute stroke MRI and corresponding chronic cognitive outcome measures.

### DICOM data selection

Identifying the specific subset of DICOM objects acquired during a scanning session is a fundamental, yet frequently neglected challenge in integrating automated analysis tools into clinical settings. To ensure the downstream success of the various algorithms in the pipeline, different image modalities (structural/anatomical sequence, DWI, and ADC) must be reliably identified in a fully automated way. For this purpose, we utilized our open-source *dcm_classifie*r package. This extensible classification framework extracts key features from well-characterized DICOM header fields to identify image modality and acquisition plane with over 99% accuracy^57^.

For the current pipeline, we extended the tool’s capabilities with post-processing functionality that encompasses several critical steps: (1) automatic selection and labeling of individual sequences including T1w, T2w, FLAIR, DWI, and ADC; (2) prioritization of axial and isotropic acquisition planes, which are standard in stroke imaging; (3) verification of sufficient image extent for complete brain coverage; (4) computation of standardized ADC images when sufficient diffusion data is available; and (5) conversion of the images into 3D volumes in NIFTI or NRRD format using ITK^58^. This fully automated procedure ensures robust input data selection in a clinical environment. Regardless of manufacturer or model, it is generalizable to any DICOM-conforming MRI scanner, making it highly scalable across different clinical settings.

### Data preprocessing

Following image selection, the preprocessing pipeline transforms raw imaging volumes into analysis-ready data through several integrated steps.

First, we classify brain versus non-brain (scalp, skull) by generating a brain mask for all imaging modalities using a custom-trained 3D ResUNet model^59^. These masks enable skull-stripping and define regions of interest for subsequent analyses. We trained brain mask models using a three-fold approach: initial model development with 3831 images, hyperparameter optimization with an independent validation set (*N* = 496 images), and final performance assessment on a separate test set (*N* = 496 images) (Supplementary Table 1). This model achieved an average Dice coefficient of 0.98 across modalities (Supplementary Table 2). Detailed descriptions of datasets and evaluation protocols are provided in the *Supplementary Material*. For quality assurance, we implemented a cross-validation framework that compares brain masks across modalities, identifying and excluding outliers through statistical analysis to optimize the anatomical accuracy of the brain masks. We then we employ the MIQA tool^60^ to systematically evaluate each image for brain coverage, artifacts, and motion corruption, generating objective quality scores.

We select the optimal input data by identifying the highest-quality image for each modality (T1w, T2w, FLAIR, ADC, and DWI) that passes the brain mask validation. The highest-scoring anatomical image is selected as the target for subsequent processing, with minimum requirements of one anatomical sequence, DWI, and ADC to proceed.

Finally, we generate a normalized, high-resolution (1 mm isotropic) T1w volume from the target image using SynthSR^61^. This synthesized volume provides standardized contrast properties, enhancing registration reliability and mitigating variability in clinical acquisitions.

### Template brain registration

Registration to the MNI-152 standard brain template enables the application of brain atlases for anatomical description and extraction of prognostic information from statistical models. While stroke lesions can potentially affect registration accuracy, our approach leverages the high-quality synthesized T1w images from the preprocessing step, effectively normalizing pathological changes and improving registration outcomes^61^. The registration process begins with co-registering the highest-quality image from each modality to the synthetic T1w derivative using sequential rigid and affine transformations implemented through ITK, with brain masks defining the registration sampling region to enhance accuracy, following methodology in BRAINSFit^62^ (Supplementary Fig. 2). We then create a transformation between this synthesized T1w image and the MNI-152 T1w template using a combination of rigid, affine, and non-linear transformations implemented through ITK^58^ and ANTs^63,64^, with brain masks defining the registration sampling region (Supplementary Fig. 3). This single transformation is then applied to all images that were co-registered to the synthesized T1w image, efficiently bringing them into MNI-152 space and resampling them to template dimensions. We performed validation testing on 2,987 individual images as described in the *Supplementary Material*, achieving a 99.7% registration success rate. Notably, the approach using the synthesized T1w image outperformed a conventional approach using the original clinical images (*Supplementary Material*), demonstrating a clear benefit to the use of synthesized T1w images for registration and thus supporting the findings in ref. ^61^.

### Lesion segmentation

Accurately segmenting acute ischemic stroke lesions is integral to the pipeline, as the lesion masks serve as the foundation for all subsequent neuroanatomical quantification and outcome-prediction models. The current implementation builds upon our previously described deep-learning lesion segmentation method^65^. The model utilizes DWI and ADC sequences as complementary input modalities; an extensive input-modality analysis demonstrated that adding T1-weighted, T2-weighted, or FLAIR sequences produced no statistically significant improvement in segmentation performance (Supplementary Table 3). Preprocessing follows the pipeline detailed in our prior work (47) and includes percentile-based intensity normalization (scaled to –1 to +1 using foreground intensities only) without hard clipping, standardization to 1 mm isotropic resolution, and resampling to a fixed matrix of 192 × 192 × 160 voxels using center-of-gravity alignment.

The segmentation model employs a 3D Residual U-Net architecture (42) that combines the localization precision of a 3D U-Net (66) with residual connections to mitigate vanishing gradients. The network consists of an encoder–decoder structure with skip connections, strided convolutions for down-sampling, transpose convolutions for up-sampling, and residual units in both pathways. Training was performed on a combined dataset of approximately 700 subjects ( ~ 450 from the Iowa retrospective clinical cohort with manual lesion delineations and all 250 subjects from the ISLES 2022 challenge dataset) using an NVIDIA RTX 8000 GPU. The Iowa portion was split at the subject level into training, validation, and held-out test sets (80/10/10); the validation set guided checkpoint selection. Post-processing converts probability maps to binary masks, followed by in-plane morphological refinement (2 × 2 voxel dilation followed by 1 × 1 erosion) to preserve anatomical coherence while respecting the original slice-wise characteristics of clinical diffusion imaging. All pre- and post-processing steps were implemented with ITK and MONAI libraries. The final model achieved a mean Dice coefficient of 0.74 on the held-out internal test set (*n* = 100). Once segmented, the acute infarct is represented as a binary 3D volume in MNI152 template space (Fig. 2).

### Evaluation of pipeline performance and segmentation

To evaluate the image processing and lesion segmentation module, we tested its performance on 160 MRIs acquired for the diagnosis and management of stroke within 7 days of the infarct. These scans are independent of the data used to train the lesion segmentation deep learning models. Measures of robustness, accuracy and runtime were all considered. To help evaluate performance, we implemented a custom pipeline profiler class that monitors execution, time, memory, and resource usage in a way that is logged for tracking. A subset of the MRIs in the test cohort was manually traced for comparison to automated lesion segmentation (*N* = 57).

### Lesion-informed cognitive outcome prediction

Our overarching goal is to utilize lesion segmentations to generate individualized predictions of post-stroke functional outcomes. To demonstrate the feasibility of this goal, we used a training dataset to develop statistical models to predict impairment on neuropsychological outcome measures based on lesion location. We applied these models to an independent test dataset to evaluate prediction performance. Lesion location was the primary input to the models; we also assessed whether performance could be improved by incorporating lesion-derived network features and demographic information (i.e., age and education). The strategy for model training and testing is summarized in Fig. 3. Inclusion criteria for both training and test cohorts included cognitive outcome data obtained for at least one of 28 cognitive outcomes, which were selected based on their representation in both the training and test datasets (Supplementary Table 6). We included cognitive assessments performed 3 months or later after the lesion onset whenever possible (98.7% of training dataset patients). The study was performed in accordance with the Declaration of Helsinki. All procedures involving human participants were approved by the University of Iowa Institutional Review Board (IRB). Data used for model training and development, as well as the independent clinical test dataset, were collected under IRB# 200105018, for which written informed consent was obtained from all participants. The test dataset was a retrospective sample of convenience pulled from electronic medical records. The need for consent was waived in accordance with the University of Iowa Institutional Review Board policies of using de-identified clinical datasets.

### Training and test datasets for cognitive outcome prediction

We identified 604 patients from the Iowa Lesion Registry that met our criteria for inclusion in the training dataset. Patients were included if they had focal, acquired brain lesions and research-quality structural neuroimaging. Patients were excluded from participation in the Registry if they had pre-existing intellectual disability or pre-existing neurological or psychiatric disorders. As mentioned, we identified 28 different cognitive assessments based on their representation in the training and testing datasets and trained classification models to predict impairment status for each of these 28 outcomes, described in detail below. Demographic details for the training dataset are provided in Supplementary Table 6. Lesion overlap maps for the training dataset are provided in Supplementary Fig. 4A.

To assess the performance of our proposed system for predicting cognitive outcomes following ischemic stroke, we compiled an independent test dataset of 153 patients with relevant neuroimaging and neuropsychological outcome data. Patients were selected from a retrospective review of clinical records at the Benton Neuropsychology Clinic (University of Iowa Healthcare System), with a confirmed ischemic stroke diagnosis between 2002 and 2023. Inclusion required: (i) clinical MRI data, acquired within one week of stroke onset, including DWI and at least one structural sequence (T1-weighted, T2-weighted, or FLAIR); and (ii) chronic-stage cognitive outcomes, assessed post-stroke via clinical neuropsychology assessment. This cohort is a subset of the 160 used to evaluate the processing and lesion segmentation pipeline. Seven individuals did not have eligible cognitive outcome data and could only be included in the imaging analysis. The imaging data represent substantial heterogeneity, derived from 17 unique scanner models across four manufacturers (Siemens, Philips, GE Medical Systems, and Olea Medical), utilizing 1.5 T and 3 T field strengths. This diversity of scanners and magnet strength mirrors real-world clinical variability to help evaluate the generalizability of our out-of-sample test results. Demographic details for the test dataset are provided in Supplementary Table 6. Lesion overlap maps for the test dataset are provided in Supplementary Fig. 4B.

### Neuropsychological outcome measures

Neuropsychological outcomes were selected on the basis of their being acquired in both the training and test datasets. This resulted in a set of 28 neuropsychological outcomes. Each outcome is listed individually along with corresponding abbreviations used in the main text and figures in Supplementary Table 7. When possible, neuropsychological scores were adjusted for age or age and education using published norms and converted to population-normed *z*-scores^66^. For each neuropsychological measure, patients were classified as either “impaired” or “not impaired”. For the MAE subtests, impairment thresholds were defined according to previously published cutoffs^67^. For all other tests with population normed *z*-scores, the threshold for determining “impaired” status was defined as a population-normed *z*-score of -1.5 or below. For outcomes corresponding to clinician ratings of impairment, which are subjective clinician ratings determined according to clinical impression and include ratings of 1 (unimpaired), 2 (borderline), and 3 (impaired), scores less than 3 were considered “not impaired”, while scores of 3 were considered “impaired”. We note that per the Benton Clinical Handbook, ratings of 2 (borderline) are defined as: “…there is some question as to whether or not the performance is normal, but it is clearly not defective”. For the Benton Test of Facial Recognition, previously published cut-off scores (i.e. corrected score < 37) were used^68^. The sample sizes for each neuropsychological measure are shown along with impairment frequencies for each measure for both the training and test datasets in Supplementary Table 8.

### Lesion segmentation and lesion-network estimation

For the training dataset, lesion masks were manually segmented and registered to the MNI-152 brain template as described previously^18^. Lesion data were resampled to 2 mm isotropic resolution. We also used the lesion location information to infer the connectivity of lesioned tissue using normative “connectome” datasets as performed previously^18,32,69,70^. We refer to the resulting lesion-derived connectivity patterns as “lesion-network maps”. Structural lesion-network maps represent the normative white-matter connections that are expected to be disrupted by the lesion, while functional lesion-network maps represent the normative functional connectivity profiles of lesioned tissue. Structural lesion-network maps were generated with Lead-DBS by using each patient’s lesion as a seed region in deterministic tractography analyses of diffusion MRI data from the Human Connectome Project’s MGH 32-fold group connectome, as reported in other publications^70^, resulting in an estimated streamline disconnection map for each patient. Functional lesion-network maps were generated analogously by using each patient’s lesion as a seed region in resting-state functional connectivity analyses in the GSP-1000 normative subject sample described previously^71^ using the principal component disconnection method^72^, resulting in an estimated lesion functional connectivity map for each patient.

For the test dataset, lesion masks were automatically generated using the lesion prediction pipeline. Lesion-network maps were not generated for the test dataset, as this functionality does not currently exist in the automated lesion prediction pipeline. Instead, lesion loads were computed for each patient as described in the next section.

### Cognitive outcome-prediction model training

We trained multivariate partial least squares (PLS) classification models to classify patients as “impaired” or “unimpaired” in terms of their performance on each of the 28 cognitive outcome measures based on their lesion location, as outlined in Fig. 3. Model training was performed using the Iowa Brain-Behavior Modeling Toolkit^32^ in MATLAB r2022b (The MathWorks). We trained partial least squares (PLS) classification models using lesion segmentations and derived lesion-network features obtained from the training dataset, and then applied these trained models to the test dataset to evaluate model performance. Hyperparameter optimization was performed using 5 repetitions of 5-fold cross-validation within the training dataset to determine the number of PLS components to include in each classification model using default settings in the Iowa Brain-Behavior Modeling Toolkit. Training and test folds for the hyper-parameter optimization were stratified by group (impaired vs. not impaired) to ensure representation of both groups in the training and test folds. As shown in Supplementary Table 7, there were large class imbalances for many of the neuropsychological outcome measures. To mitigate this during model training, we set the misclassification costs for patients with impairment proportional to the class imbalance (e.g., if there were twice as many patients without impairment, then the misclassification cost for patients with impairment was set to twice that of patients without impairment). The final classification score thresholds for each outcome were then tuned using ROC curves obtained from the training dataset, along with the misclassification cost matrix, to identify the threshold that optimized class separation in the training dataset.

For each outcome, we generated one lesion location model, one structural lesion-network model, and one functional lesion-network model using the training dataset. The lesion location models were trained using voxel-based lesion masks as predictor features, while the structural and functional lesion-network models were trained using the derived structural and functional lesion-network maps. For models using structural and functional lesion-network maps, the input features were standardized to the range [0,1] and [-1,1], respectively, using centering and scaling factors defined in the training dataset. This resulted in three voxel-based PLS model coefficient maps for each outcome, one for the lesion location model, one for the structural lesion-network model, and one for the functional lesion-network model.

The automated lesion segmentation pipeline does not currently include the functionality required to generate lesion-derived network features, and so we could not directly apply the trained structural lesion-network and functional lesion-network models in the test dataset. To work around this limitation, we trained a second set of models, again in the training dataset, using lesion loads computed against the PLS model coefficient maps generated by the initial lesion-network models in the training dataset. Lesion loads on each lesion-network map were computed for each patient in the training dataset by taking the dot product between the patient's lesion segmentations and the lesion-network model PLS coefficient map.

Finally, we trained “stacked” ridge-regularized logistic regression models that used the predicted class labels (impaired vs. unimpaired) from the lesion location model, the lesion loads on the structural lesion-network model coefficient map, and the lesion loads on the functional lesion-network model coefficient map as predictors. All features in the “stacked models” were standardized to range [0,1]. These “stacked” models, therefore, incorporated information about lesion location along with associated structural and functional networks. We note that while training secondary models using the lesion loads computed against the model coefficient maps generated from the training datasets is an inherently circular analysis within the training dataset, it should not bias evaluations of model performance in the fully independent test dataset since the test data were not used for generating or training either model. We also evaluated the impact of adding age and education information to the stacked models. For these analyses that included demographics, 36 training dataset patients and 10 test dataset patients were excluded due to missing education data.

### Cognitive outcome-prediction model evaluation

All models were trained in the training dataset and applied to the independent test dataset. Cross-validation was also performed in the training dataset, as described above. For estimates of cross-validation performance of the stacked models obtained using the training set, lesion loads were computed using training fold coefficient maps (i.e. instead of the group-level coefficient maps) that were generated independently from the test folds, mirroring the approach used in the application to the independent test dataset and avoiding circularity and the introduction of biases into the cross-validation estimates. Cross-validation results obtained from the training dataset are provided in Supplementary Fig. 5.

Each trained single-modality model was applied directly to the test dataset. For the lesion location models, the trained models were directly applied to the automatically generated lesion masks from the test dataset. For the models incorporating lesion loads on the lesion-network model PLS coefficient maps, input features for the test dataset were computed in the same way as for the training dataset (i.e., by taking the dot product between each test set patient’s vectorized lesion mask and the vectorized model coefficient map from the training dataset). For each neuropsychological test, this generated 4 predictions for each patient in the test dataset: one from the lesion location model, one from the structural lesion-network lesion load model, one from the functional lesion-network lesion load model, and one from the “stacked” model that combined the predicted class labels from the lesion location model with the lesion loads computed from the structural lesion-network and functional lesion-network model coefficient maps (and for the secondary analyses, age and education variables).

Model performance is reported in terms of the area under the ROC curve (AUC), which is robust even in the presence of large class imbalances such as those observed in the datasets under study, in line with recommendations for measurement of classification accuracy in human neuroimaging studies^73^. For descriptive purposes, we also report the classification accuracy for impaired patients and the classification accuracy for unimpaired patients using the tuned classification score thresholds that optimized class separation in the training dataset for the 5 select models shown in Fig. 5. To evaluate the statistical significance of the observed classification accuracies at the trained thresholds, we performed permutation tests (1,000 permutation iterations) for each model and each outcome. These tests involved randomly shuffling the predicted group labels and then recomputing the AUCs to construct an empirical null distribution of AUC estimates. The observed classification accuracy was then compared against the empirical null distribution to obtain a *p*-value indicating the probability of obtaining an AUC at least as extreme as the observed value under the null hypothesis that the predicted classification scores contain no information about impairment status. Finally, we compared overall performance (i.e., test dataset AUCs across all 28 outcome measures) between the lesion-only models and stacked models using two-tailed Wilcoxon signed-rank tests.

We also performed additional analyses to evaluate the comparability of cognitive outcome model predictions obtained in the test set when using manual vs. automatically generated lesion segmentations. For a subset of test dataset patients that also had manually segmented lesion images (N = 57), the trained lesion-only models were applied to both the manual lesion segmentations and the automated pipeline lesion segmentations to obtain two sets of predicted class labels – one for the manual lesion segmentations and one for the automated lesion segmentations. This resulted in a total of 681 predictions per lesion segmentation method across the 28 different neuropsychological outcome measures. The concordance between the predicted class labels obtained from the automated vs. manual lesion segmentations was then measured for each neuropsychological outcome and across all outcomes simultaneously. The results of these analyses are reported in Supplementary Fig. 6.

### Report generation

Reports are generated as PDF files using ReportLab and Meta’s LLaMA 3.3 (70B) for narrative synthesis, combining templated text with imaging-derived insights (e.g., lesion volume, location). The reports synthesize neuroimaging analyses into documentation that could be utilized by clinicians. Clinician-focused sections include an overview, radiological analysis, neuroanatomical analysis, and outcome prediction. The radiological analysis provides visualizations of DWI and ADC sequences with lesion segmentation overlays, alongside 3D renderings in MNI space for spatial context. Visualizations utilized ITK and VTK libraries.

The neuroanatomical analysis integrates two complementary brain atlases for comprehensive lesion characterization: an arterial territory atlas defining 30 vascular regions (modified from Liu’s arterial supply atlas) and an anatomical atlas parcellating the brain into 154 distinct regions. The anatomical atlas builds upon the Harvard-Oxford Cortical and Subcortical Atlases, incorporates the Morel Thalamic Atlas for enhanced thalamic detail, derives brainstem regions from FreeSurfer v6 processing of the MNI152 template, uses the SUIT atlas for cerebellar parcellation, and defines white-matter territories (including corpus callosum and cerebral/cerebellar tracts) from a population-level tractography atlas. Using inverse transformation matrices computed during the registration pipeline, these atlases are warped into each patient’s native image space via affine and deformable transformations. Lesion overlap is then quantified using a voxel-wise approach on binary masks, yielding three key metrics for each affected structure: absolute lesion volume in cubic centimeters, percentage of total lesion volume within the region, and percentage of each anatomical region affected by the lesion (partial volume effects addressed through proportional allocation). Results are presented in structured hierarchical tables organized across three levels (hemisphere, cortical anatomical regions/lobes or arterial territories, and subcortical structures). This framework enables rapid assessment of macroscopic damage distribution, white-matter versus gray-matter involvement (for disconnection syndromes), and vascular territory contributions .

Outcome predictions, personalized for each patient, are displayed as binary (low or high) risk categories for different cognitive domains, enabling rapid assessment of functional impairment risks.

The final two report pages are tailored for patients and families, translating complex medical findings into accessible, non-technical language. These sections cover the stroke event, expected outcomes, risks, and actionable steps, using relatable analogies and avoiding medical jargon. Readability is optimized for a 6th–7th grade level (SMOG metric: mean 6.6, max 7.8, SD 0.6), ensuring comprehension across diverse literacy levels and supporting patient engagement in recovery planning.

To mitigate the well-documented risks of hallucinations and factual drift when applying large language models to clinical text synthesis^74^, we implemented a multi-layered guardrail framework. The LLM (Meta’s LLaMA 3.3 70B) is hosted locally via Ollama on an isolated server with no internet connectivity. This air-gapped deployment ensures complete data isolation, eliminates any risk of external transmission or leakage, and maintains HIPAA compatibility. The model receives exclusively a fully de-identified, structured data object generated by the pipeline; it never accesses protected health information, patient identifiers, raw images, or free-text clinical notes. The system is deliberately stateless between queries, with no retention of conversation history or memory across report sections, providing a clean context start for every template and further reducing cross-referencing errors.

The majority of each report is constructed from fixed templates, with the LLM’s role strictly limited to natural-language reorganization and readability enhancement within narrowly scoped, section-specific prompts. Each clinician-facing and patient-facing section operates independently to eliminate context-bleed risks. Prompts explicitly prohibit generation of new clinical insights, treatment recommendations, or any content not directly supported by the input data fields. LLM outputs are automatically validated post-generation by a custom Markdown parser that enforces template adherence and flags structural deviations. Finally, every report underwent a technical review. No instances of hallucination or structural drift were identified in reports from the independent test dataset (*N* = 153). This constrained, privacy-first approach aligns with emerging best practices for safe clinical LLM deployment in medical safety-critical settings^75^.

### System configuration

The system is engineered for seamless integration into clinical workflows. It receives raw DICOM data from hospital PACS, processes the images through the automated pipeline, and returns DICOM-encapsulated reports that appear directly alongside the original imaging studies in radiological workstations. These reports are initially generated as PDFs using a modular architecture based on the open-source ReportLab library and then converted to DICOM format with appropriate study, series, and instance metadata, ensuring longitudinal availability and security within existing hospital infrastructure. The platform operates as a containerized application using Docker. Narrative summaries are produced by a locally hosted large language model (LLM) server running in a separate Docker container using Ollama. This LLM instance receives only de-identified, extracted features from the pipeline and never has access to protected health information, thereby maintaining full HIPAA compatibility.

## Supplementary information

Supplementary Information

## Data Availability

The software used for lesion modeling can be downloaded at the provided URL. Deidentified data from this study may be made available upon reasonable request to the corresponding author, subject to a data use agreement ensuring compliance with human subjects ethical standards. Trained machine learning models for outcome prediction may be shared, subject to a proper software use agreement protecting intellectual property rights. The IBB toolkit version used for training and evaluating the cognitive outcome-prediction models is archived along with the notebooks used for generating the outcome-prediction figures here: https://zenodo.org/records/15626633. https://zenodo.org/records/15610144. The dcm-classifier package used for DICOM modality classification is available as PIP package at https://pypi.org/project/dcm-classifier/.
