# Supplementary material for: A clinical neuroimaging platform for rapid, automated lesion detection and personalized post-stroke outcome prediction
Source: NPJ Digit Med. 2026 May 27;9:646. doi: 10.1038/s41746-026-02803-2 (PMC13493867; doi:10.1038/s41746-026-02803-2)
Supplement: Supplementary file 1 [file 41746_2026_2803_MOESM1_ESM.pdf]

Supplementary Materials

Automated Image Processing Pipeline

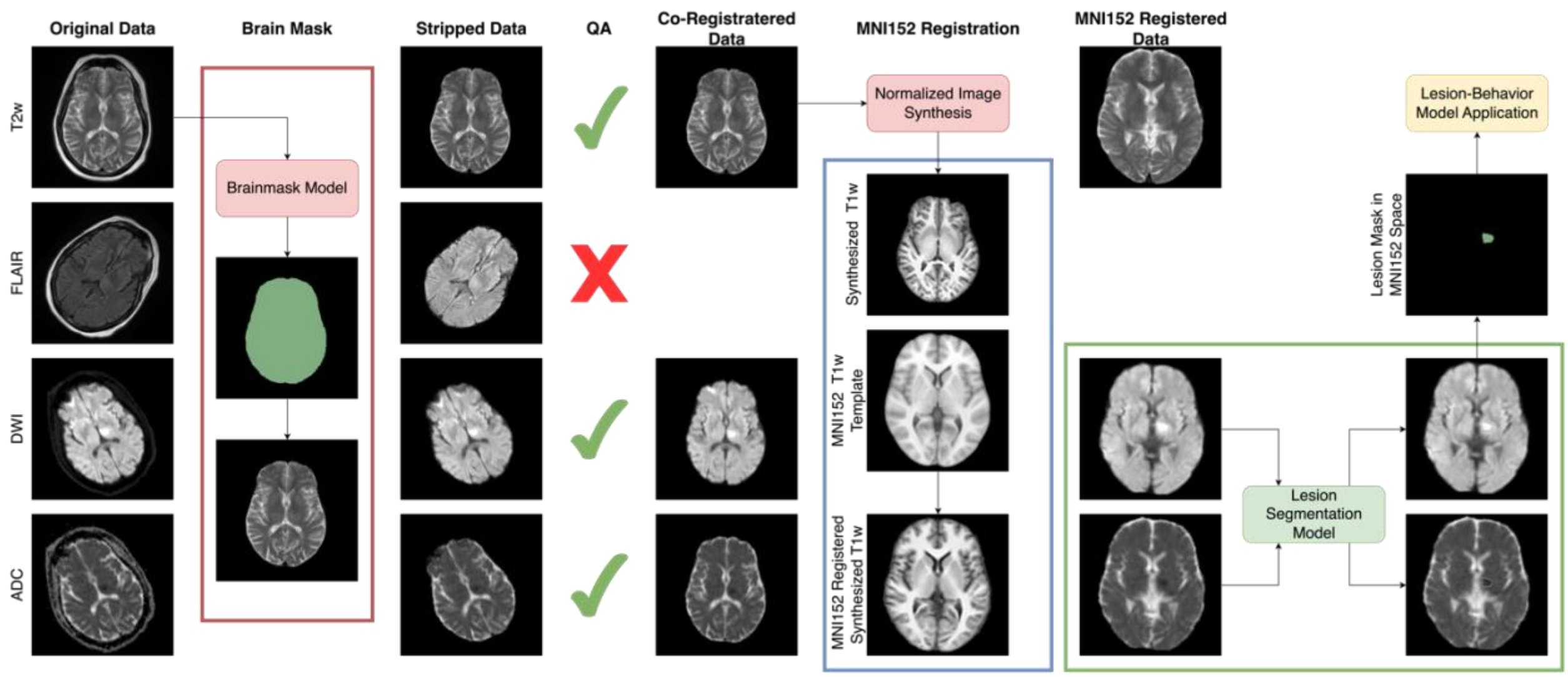

Supplemental Figure 1. Visualization of brain MRI processing steps from raw clinical image to segmented lesion masks in MNI152 template space for lesion-behavior model application.

Development Datasets

Iowa Dataset. For the development and validation of the automated processing pipeline, we utilized a retrospective dataset of 1,047 ischemic stroke patients from the University of Iowa Hospital and Clinics (UIHC) archives (IRB-approved and anonymized). This dataset comprised standard-of-care MRI scans acquired within one week of stroke onset, spanning from December 2009 to October 2021. The imaging data exhibited substantial technical heterogeneity, having been collected across 20 unique scanner models from 5 major manufacturers (Siemens, Philips, GE, Toshiba, and Hitachi). This diversity of acquisition sources intentionally introduced technical variability that strengthened the generalizability of our pipeline to heterogeneous clinical environments.

Different subsets of this dataset were strategically employed for various pipeline components: fine-tuning the DICOM classifier tool, developing and validating brain mask models, training lesion segmentation algorithms, and optimizing registration approaches. The extensive temporal range and diversity of acquisition parameters enhanced the robustness of our models to clinical variability.

ISLES 2022 Dataset. The ISLES 2022 dataset was obtained from the Ischemic Stroke Lesion Segmentation Challenge (ISLES22) *(1)* and contains 250 subjects with scans of varying quality. This dataset was designed specifically for the evaluation of automated stroke lesion segmentation methods and represents a multi-center collection of clinical stroke MRI data. The challenge

dataset includes multi-modal MRI sequences (DWI, ADC, FLAIR) acquired in acute stroke settings across multiple international medical centers, capturing a wide range of lesion sizes, locations, and imaging protocols. Unlike curated research datasets, ISLES 2022 intentionally incorporates the real-world variability found in clinical environments, including motion artifacts, varying field strengths, and diverse acquisition parameters. This heterogeneity makes it particularly valuable for validating the robustness and generalizability of automated stroke processing pipelines in realistic clinical scenarios.

Brain Mask Dataset. The brain mask model training dataset was derived from the Iowa dataset described above. As depicted in Supplementary Table 1, we utilized 4,823 images from 1,047 patients across five MRI modalities: T1-weighted (T1w), T2-weighted (T2w), Fluid-Attenuated Inversion Recovery (FLAIR), and Diffusion Weighted Imaging derivatives—Apparent Diffusivity Coefficient (ADC) and Diffusion Trace (DWI).

The Ground Truth masks used for training the brainmask model were initially generated using the SynthStrip algorithm. Each mask was then manually verified and updated where needed to ensure accuracy. While SynthStrip generally performs well, we identified failure cases, particularly with diffusion images, motivating the development of a custom model.

Supplementary Table 1. Brain Mask Datasets

Modality	Training	Validation	Testing	Total
T1w	754	101	98	958
T2w	577	79	85	741
ADC	839	106	106	1051
Flair	817	104	101	1022
DWI	839	106	106	1051
**Total**	**= 3831**	**= 496**	**= 496**	**= 4283**

Image Processing Pipeline – Supplementary Results

Brain Mask. The automated brain extraction model demonstrated remarkable consistency across multiple MRI modalities. We employed a three-fold approach to model development: initial training with 3,831 images from 837 subjects, hyperparameter optimization with an independent validation set of 496 images from 104 subjects, and final performance assessment on a separate test set of 496 images from 106 subjects. This corresponded to an approximate 80:10:10 percent split at the subject level, ensuring that images from the same subject never appeared across different datasets to prevent data leakage. The model was evaluated on five different sequences commonly used in stroke imaging: T1-weighted, T2-weighted, FLAIR, ADC, and DWI. On the test dataset, the model achieved a mean DICE score of 0.98 across all modalities, with notably low variability (standard deviation ≤ 0.01 for all sequences). Boundary accuracy, measured by balanced Average Hausdorff Distance (bAVD), was also highly consistent, ranging from 0.16 to 0.24 voxels across modalities (Supplementary Table 2). This stability across different imaging

contrasts is particularly important for robust preprocessing in clinical settings where multiple MRI sequences are routinely acquired.

Supplementary Table 2. Brain Mask Results

Modality	DICE		bAVD	
	Mean	Std	Mean	Std
T1w	0.98	0.00	0.16	0.00
T2w	0.98	0.00	0.16	0.00
ADC	0.98	0.01	0.24	0.01
Flair	0.98	0.01	0.17	0.01
DWI	0.98	0.00	0.16	0.01

Registration. We validated our registration approach on a comprehensive dataset of 2,987 clinical MRI scans derived from the Iowa dataset and comprising 1,122 FLAIR images (37.6%), 1,041 T1-weighted images (34.9%), and 824 T2-weighted images (27.6%) from diverse acquisition protocols, scanner manufacturers, and field strengths. To assess the effectiveness of our synthetic image-based approach, we conducted a comparative analysis between conventional registration using original clinical images and our synthetic pipeline using SynthSR-generated T1w images. Both pipelines employed identical registration parameters, with evaluation metrics calculated on the original clinical images after transformation to ensure fair comparison.

The synthetic image-based approach demonstrated clear advantages in both accuracy and robustness. The synthetic approach achieved consistently higher DICE scores between registered brain masks and the MNI template mask (0.960 vs. 0.942) across all imaging modalities. Most notably, while the conventional pipeline resulted in 44 failures out of 2,987 cases (98.5% success rate), the synthetic pipeline reduced this to only 8 failures (99.7% success rate). This exceptional reliability has significant implications for clinical deployment, minimizing the need for time-consuming manual corrections while enabling successful processing of cases that would typically fail in conventional pipelines. The synthetic approach's consistent performance across varying input data quality is particularly valuable in clinical settings where imaging protocols may vary considerably, confirming its suitability for routine clinical deployment.

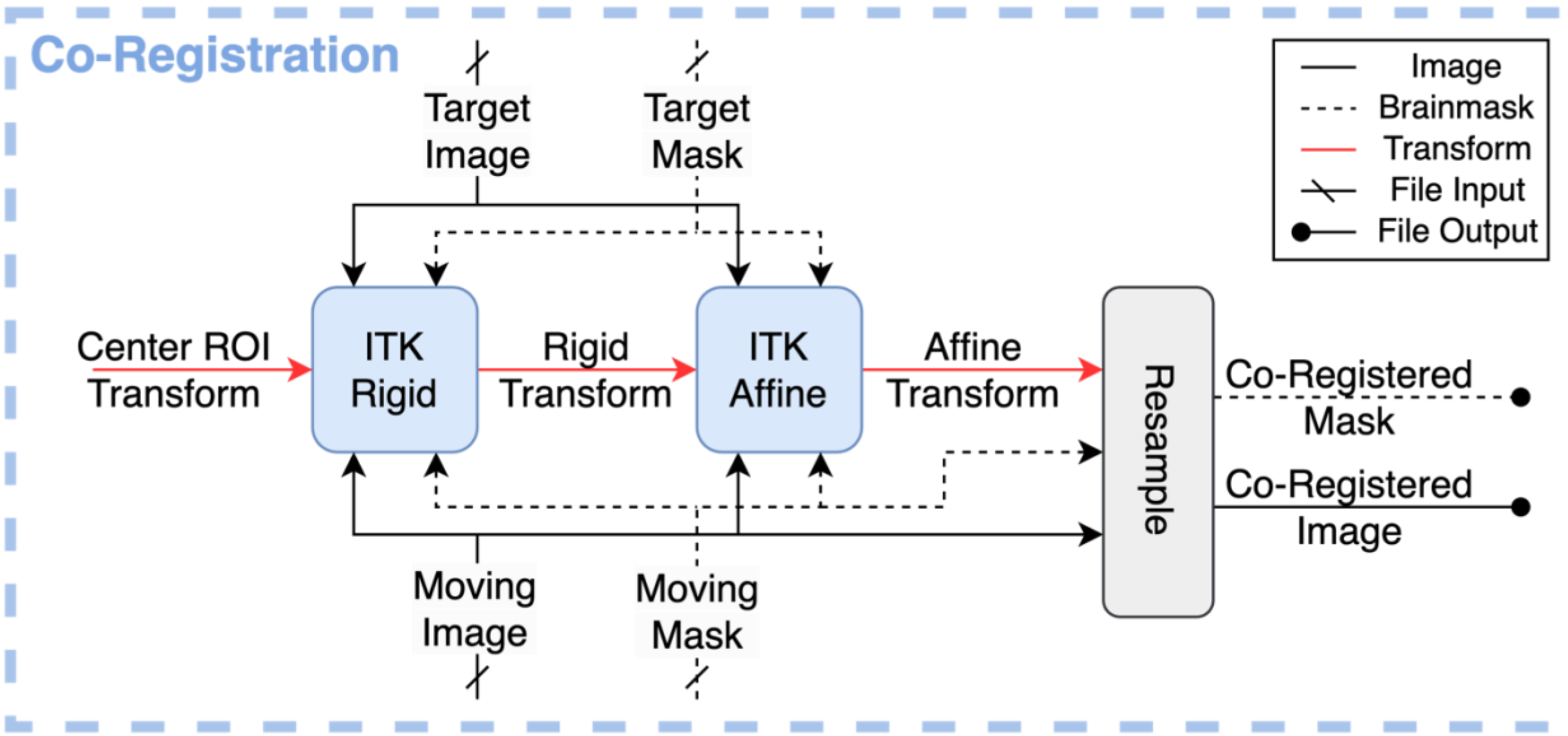

Supplementary Figure 2. Co-registration pipeline utilizing sequential rigid and affine registration steps. The process employs brain masks to guide registration metrics, with the target image serving as the fixed reference while remaining modalities are treated as moving images.

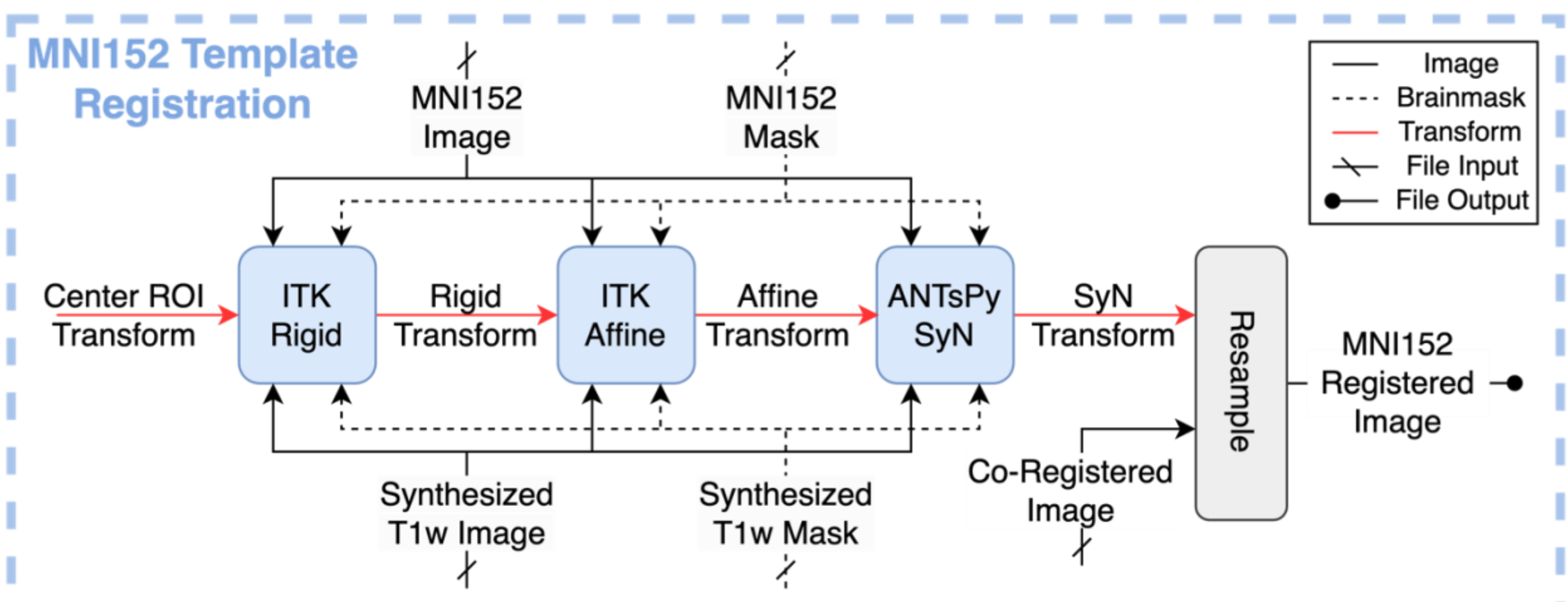

Supplementary Figure 3. Simplified View of the Template Registration module showing the three-stage registration process that transforms patient-specific MRI data into standardized MNI152 space.

Lesion Segmentation: Input Modality Analysis. To determine the optimal input modality combination, we conducted a comparative analysis of different MRI sequence combinations for lesion segmentation. Four identical 3D Residual U-Net models were trained using the same subset of the Iowa Stroke Retrospective Dataset (342 subjects containing all five modalities of interest: DWI, ADC, T1w, T2w, and FLAIR), with consistent data splits across all configurations (80% training, 10% validation, 10% testing). These models varied only in their input modalities: (1) DWI+ADC only, (2) DWI+ADC+T1w, (3) DWI+ADC+T2w, and (4) DWI+ADC+FLAIR.

As shown in Supplementary Table 5, adding anatomical sequences provided no statistically significant improvement over diffusion-weighted images alone. The baseline model (DWI+ADC) achieved a mean DICE score of 0.792, comparable to models incorporating additional anatomical sequences (DICE 0.788-0.799). This finding aligns with clinical understanding that acute stroke lesions are often not visible on structural images, being primarily detectable on diffusion-weighted sequences. The absence of meaningful performance gains, coupled with considerations of data availability, computational efficiency, and model stability, strongly supported our decision to use only diffusion-weighted sequences in the final implementation. This streamlined two-modality approach offers practical advantages including reduced preprocessing time, smaller model footprint, and improved system efficiency without sacrificing segmentation accuracy.

Supplementary Table 3. Lesion Segmentation Input Modalities Analysis

Input Modalities	DICE Mean	DICE Std	bAVD Mean	BAVD Std
DWI + ADC (Baseline)	0.79	0.2	0.70	1.30
DWI + ADC + T1w	0.80	0.2	0.92	3.02
DWI + ADC + T2w	0.79	0.20	0.58	1.08
DWI + ADC + FLAIR	0.79	0.21	3.03	9.60

Lesion Segmentation: Test Dataset Segmentation Results. The automated lesion segmentation model was evaluated on an independent clinical test dataset comprising 57 subjects with expert-validated lesion labels. As shown in Table 4. the model achieved a mean DICE score of 0.69 across all cases, with a standard deviation of 0.21. After excluding two failed cases, performance improved to a mean DICE of 0.71 with reduced variability (standard deviation of 0.16). The balanced Average Hausdorff Distance (bAVD) of 0.95 voxels indicated strong boundary agreement between predicted and ground truth segmentations.

Supplementary Table 4. Lesion Segmentation Results

Metric	DICE	bAVD
Mean	0.69	0.95
Std	0.21	1.48

Lesion Segmentation: Temporal analysis. Temporal analysis of model performance revealed a clear trend correlated with image acquisition periods, with results shown in Table 5. The dataset spanned over two decades (2001-2024), allowing for evaluation across different eras of MRI technology. The model achieved a DICE score of 0.74 on recent data (post-2015), compared to 0.68 for cases from 2011-2015 and 0.66 for pre-2011 cases. This improvement is particularly noteworthy given that recent cases had smaller average lesion volumes (47.6 cm^3 post-2015 vs 54.1 cm^3 pre-2011), as DICE scores typically favor larger lesions (Raina et al., 2023). The consistent improvement across time periods, achieved despite this inherent DICE bias, suggests that the model effectively leverages the enhanced image quality of modern MRI scanners.

Supplementary Table 5. Lesion Segmentation Results by Time Period

Period	n	Avg. Lesion Vol. (cm^3)	DICE
Before 2011	9	54.1	0.66
2011-2015	39	40.5	0.68
After 2015	9	47.6	0.74

Lesion Segmentation: Lesion Detection Analysis. We conducted a lesion-wise detection analysis on our test dataset comprising 57 subjects with 112 individual lesions. For clarity, each connected component (individual island) in the binary lesion mask was counted as a separate lesion, which explains the multiple lesions per patient. Successful detection was defined as any overlap between the model's prediction and the ground truth lesion mask. The model demonstrated an overall detection accuracy of 0.81 across all lesions. Excluding the small lesions under 1 cm^3, accuracy improved markedly to 0.93 for lesions $\geq$ 1 cm^3 and 0.98 for lesions $\geq$ 2.5 cm^3. When excluding two previously identified failed cases (analyzing 106 lesions across 55 subjects), detection rates further improved to 0.88, 0.98, and 1.00 respectively for all lesions, lesions $\geq$ 1 cm^3, and lesions $\geq$ 2.5 cm^3.

Report Generation: Readability Analysis of Patient Communication. We conducted systematic readability analysis of our patient-directed content using the Simple Measure of Gobbledygook (SMOG) formula *(2)*, specifically chosen for its established use in evaluating health education materials. The SMOG index estimates the years of education needed to understand text by analyzing polysyllabic words within a sample of 30 sentences, providing a validated grade-level assessment. We randomly selected 100 subjects from our test set and analyzed their automatically generated patient communication sections, which are organized into four strategic sections: "What Happened," "What to Expect," "Your Risks," and "What to Do." These sections translate complex medical information into accessible language using relatable analogies and clear explanations, deliberately avoiding medical jargon. The SMOG analysis demonstrated consistent achievement of appropriate reading levels with a mean grade level of 6.6 (SD=0.6), maximum of 7.8, minimum of 4.9, and 95% confidence interval of 6.5-6.7. These results confirm that our patient-focused content consistently performs below the average US adult reading level (approximately 7th-8th grade), making the information accessible regardless of educational background and addressing the documented health literacy challenges faced by approximately 36% of US adults with basic or below basic health literacy skills *(3)*.

Cognitive Outcome Prediction

Demographic Data for Cognitive Outcome Prediction Datasets

Demographic data for the training and test datasets used for the cognitive outcome prediction analyses are provided in **Supplementary Table 6**. Data are reported as Mean (Standard Deviation) for all continuous measures. For Gender, data are reported as the total number of Male patients in each dataset. For Race, breakdowns of each dataset by race are provided. For Lesion Laterality, breakdowns of each dataset by lesion laterality are provided.

Supplementary Table 6. Demographics for outcome prediction datasets

Dataset	Age	Gender	Handedness	Education (Years)	Race	Lesion Laterality	Lesion Volume (2mm isotropic)	Time from Onset to Testing (Years)
**Train (N=604)**	53.63 (14.7)	328 Male	82.51 (50.18)	13.38 (2.80)	579 White, 6 African American, 2 Native American, 3 Other, 14 Unknown	274 Left, 212 Right, 118 Bilateral	5690.3 (8084.7)	1.36 (2.33)
**Test (N=153)**	56.27 (13.25)	79 Male	80.47 (51. 70)	13.82 (2.48)	146 White, 1 African American, 2 Native American, 2 Other, 2 Unknown	67 Left, 61 Right, 25 Bilateral	6241.5 (7466.3)	0.95 (1.45)

Neuropsychological Outcome Measures

The full set of 28 neuropsychological outcome measures is provided in **Supplementary Table 7**. Each outcome measure is listed along with the corresponding abbreviation used in the main text and figures.

Supplementary Table 7. Neuropsychological Outcome Measures and Abbreviations

1. WAIS Arithmetic (**arith**)
2. WAIS Similarities (**sim**)
3. WAIS Coding (**cd**)
4. WAIS Matrix Reasoning (**mr**)
5. WAIS Digit Span (**ds**)
6. WAIS Block Design (**bd**)
7. Chapman Reading Test (**chapman**)
8. Rey Complex Figure Test Recall (**cftr**)
9. Rey AVLT Trial 5 (**rey5**)
10. Rey AVLT Delayed Recall (**rey-dr**)
11. Rey AVLT Recognition Hits (**rey-hit**)
12. Trails A (**trails-a**)
13. Trails B (**trails-b**)
14. MAE Controlled Oral Word Association Test (**mae-cowa**)
15. MAE Sentence Repetition (**mae-sr**)
16. MAE Token Test (**mae-token**)
17. MAE Visual Naming (**mae-vn**)
18. MAE Aural (**mae-aural**)
19. MAE Reading (**mae-read**)
20. Boston Naming Test (**bnt**)
21. Benton Visual Retention Test (**brtc**)
22. Benton Test of Facial Recognition (**face-rec**)
23. Clinical Articulation Ratings (**clin-artic**)

24. Clinical Paraphasia Ratings (**clin-paraph**)
25. Clinical Fluency Ratings (**clin-fluen**)
26. Clinical Prosody Ratings (**clin-prosod**)
27. WRAT Reading (**wrat-read**)
28. Judgment of Line Orientation (**jlo**)

Neuropsychological Test Sample Sizes and Impairment Frequencies

The training and test dataset sample sizes, along with the proportion of patients with scores qualifying as "impaired" in each dataset, are provided for each individual neuropsychological test in **Supplementary Table 8.** Neuropsychological test name abbreviations correspond to those provided in **Supplementary Table 7**.

Supplementary Table 8. Sample Sizes and Proportion Impaired

	train_n	train_impaired	train_proportion_impaired	test_n	test_impaired	test_proportion_impaired
arith	517	45	0.09	92	7	0.08
bd	545	42	0.08	111	4	0.04
bnt	461	91	0.2	109	24	0.22
bvrtc	527	89	0.17	101	16	0.16
cd	137	14	0.1	95	3	0.03
cftr	543	126	0.23	135	39	0.29
chapman	288	121	0.42	49	18	0.37
clin_artic	547	41	0.07	108	5	0.05
clin_fluen	547	39	0.07	108	17	0.16
clin_paraph	546	63	0.12	106	19	0.18
clin_prosod	546	45	0.08	107	7	0.07
ds	522	49	0.09	129	15	0.12
face_cat	493	62	0.13	80	8	0.1
jlo	456	40	0.09	97	11	0.11
mae_aural	244	32	0.13	26	6	0.23
mae_cowa	557	123	0.22	132	77	0.58
mae_read	249	30	0.12	28	7	0.25
mae_sr	311	64	0.21	63	27	0.43
mae_token	380	65	0.17	59	17	0.29
mae_vn	261	38	0.15	20	6	0.3
mr	343	14	0.04	96	7	0.07
rey5	528	113	0.21	127	30	0.24
rey_dr	528	107	0.2	127	29	0.23
rey_hit	517	84	0.16	124	36	0.29
sim	486	19	0.04	104	6	0.06
trails_a	429	89	0.21	134	17	0.13
trails_b	418	97	0.23	125	19	0.15
wrat_read	384	37	0.1	106	7	0.07

Lesion Frequencies for Cognitive Outcome Prediction Datasets

Lesion overlap maps for the training and test datasets used for the cognitive outcome prediction analyses are shown in **Supplementary Figure 4**.

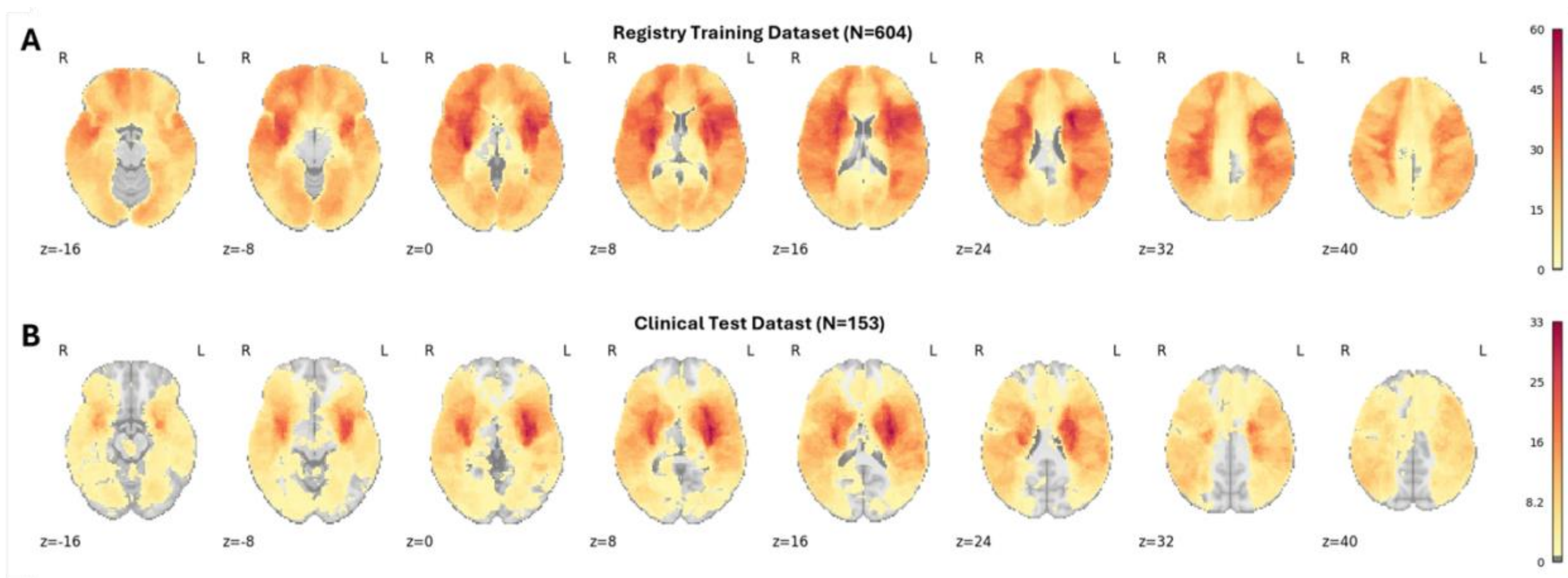

Supplementary Figure 4. Lesion Overlap Maps. **A**. Lesion overlaps for the training dataset. **B.** Lesion overlap maps for the test dataset. Voxel colors indicate the number of overlapping lesions at each voxel.

Training Dataset Cognitive Outcome Prediction Cross-Validation Results

The results of cross-validation analyses within the training dataset are shown in **Supplementary Figure 5**.

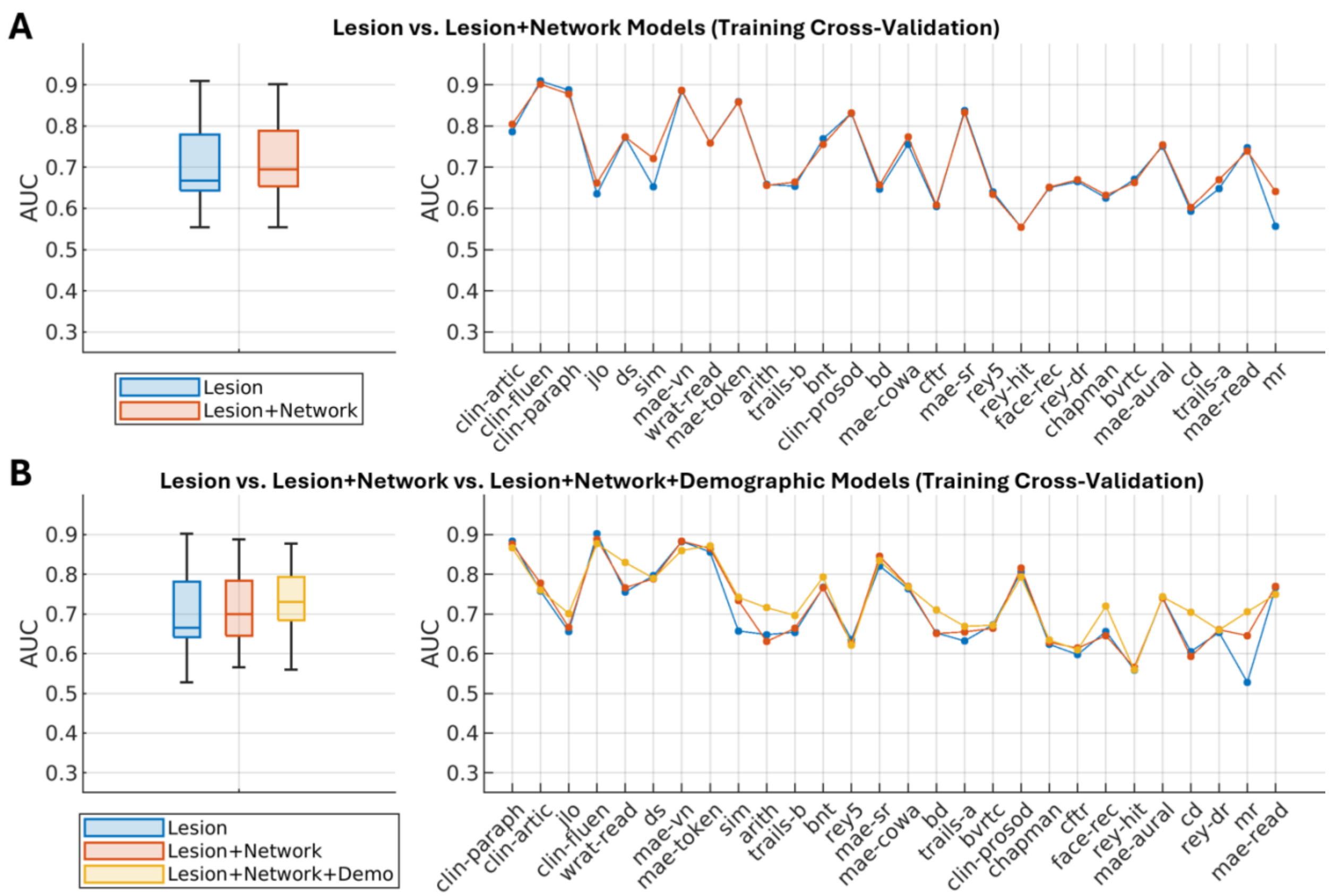

Supplementary Figure 5. Cross-validation performance in the training dataset. **A.** Cross-validation performance for 5 repetitions of 5-fold cross-validation is shown for the lesion models and lesion + network models in the training dataset. AUC values correspond to the average cross-validation test set AUC across folds and repeats of the cross-validation procedure. Cognitive outcomes in the right panel are sorted identically to **Figure 4A**, highlighting differences in relative performance in training dataset cross-validation vs. out-of-sample prediction in the independent test dataset. The rank correlation between the lesion + network model AUCs obtained from training dataset cross-validation and the lesion + network AUCs obtained from the independent test dataset was moderate (r=0.56). **B.** Same as (A), but with the addition of lesion + network + demographic models. The rank correlation between the lesion + network model AUCs obtained from training dataset cross-validation and the lesion + network AUCs obtained from the independent test dataset was moderate (r=0.53). Cognitive outcomes are sorted identically to **Figure 4C**.

Concordance of Predictions for Manual and Automated Lesion Segmentations

The concordance of predictions between manual and automated lesion segmentations is shown in **Supplementary Figure 6**. A total of 681 predictions were compared.

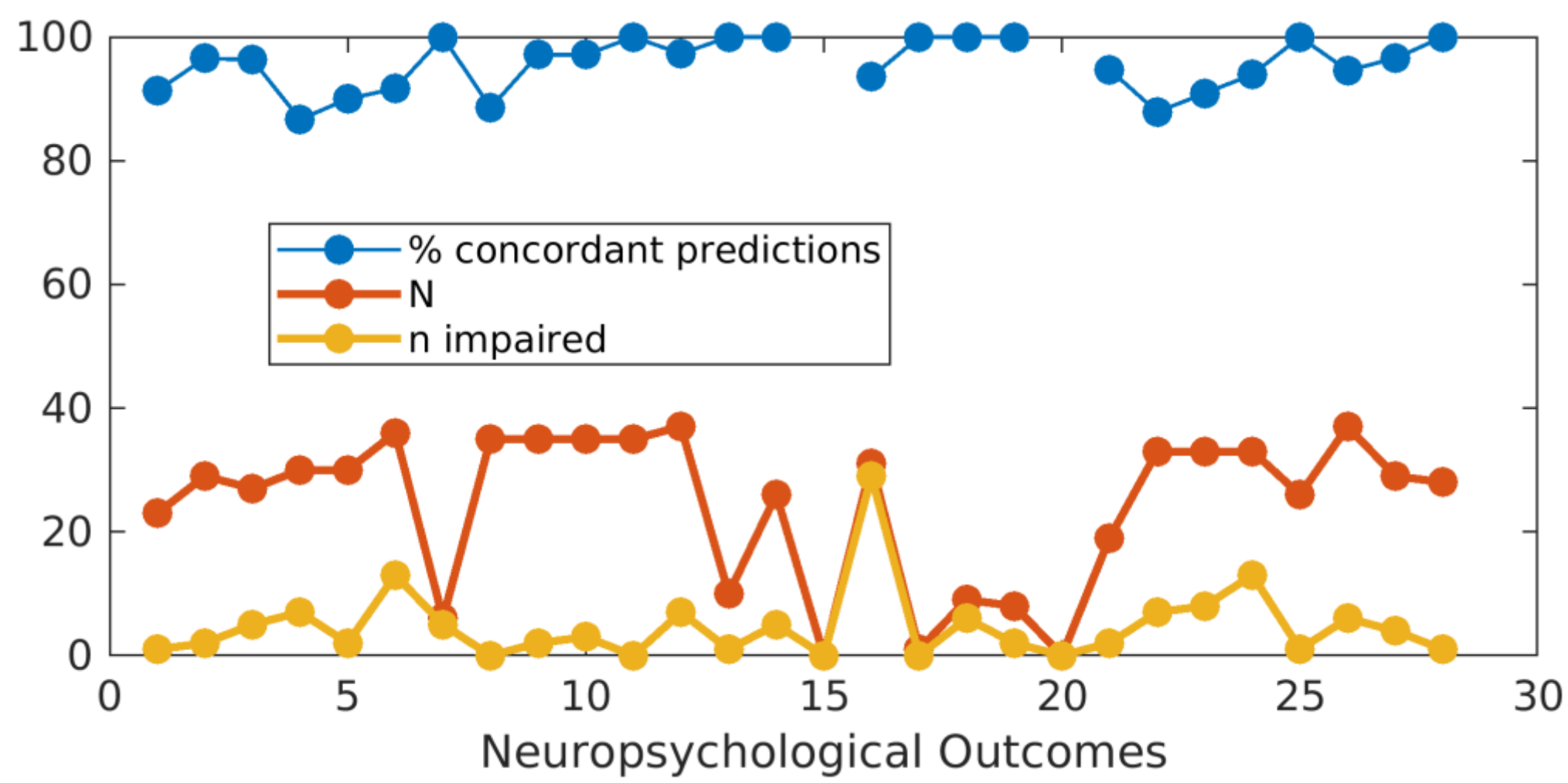

Supplementary Figure 6. The percentage of predictions that were concordant between the manual and automated lesion segmentations is shown for each outcome (blue line) along with the sample size of patients with ground truth lesions (orange) and the number of patients with ground truth lesions with impairment for each outcome (yellow). Across all outcomes, the average concordance rate was 96% (SD=4%), with a range of 87%-100%. Across all aggregated predictions (i.e. across all outcomes; 549 total predictions) for unimpaired patients, the concordance rate was 95%. Across all aggregated predictions (i.e. across all outcomes; 132 total predictions) for impaired patients, the concordance rate was 94%. These results show that for the 57 test dataset patients with ground truth manual lesion segmentations, the predicted class labels

were highly consistent regardless of whether the ground truth manual lesion segmentations vs. automated pipeline lesion segmentations were used to obtain the predictions.

System Evaluation

Runtime Analysis. We report results from a single, fully automated processing run on an out-of-sample clinical test dataset comprising 160 stroke patients. All cases met minimum eligibility criteria (one structural image, DWI, and ADC), and the system successfully processed all subjects end-to-end without triggering any fail-safe mechanisms. All intermediate outputs (image classification, brain masks, registrations, lesion segmentations) were visually reviewed for quality assessment.

Performance benchmarks were conducted on a workstation with an Intel Xeon w7-3545 CPU (24 cores, 48 threads, 4.8GHz max frequency). Most components ran on a single CPU core, except registration, which utilized 24 cores due to its computational intensity. As shown in **Table 1**, CPU-only processing achieved an average end-to-end time of 274.8 seconds (approximately 4.6 minutes), ranging from 188.1 to 472.5 seconds (SD=47.0). Registration was the most time-intensive step at 85.7 seconds on average, followed by report generation at 146.3 seconds, reflecting the sequential nature of the pipeline on the CPU. Enabling GPU acceleration on an NVIDIA RTX 6000 Ada (48GB memory) reduced the average end-to-end processing time by 55.9% to 121.3 seconds (approximately 2 minutes), with 95% of cases completing in under 3 minutes (range=80.4 to 238.1 seconds, SD=22.3). Registration time decreased by 10.5% to 76.7 seconds, while report generation saw the most significant improvement, dropping 93% to 10.0 seconds, mainly due to GPU acceleration of large language model inference for text generation. This efficiency, detailed in **Table 1**, underscores the GPU's value for rapid analysis in acute stroke care settings.

Supplementary Table 8. Runtime summary of processing system components in seconds.

System Component	CPU				GPU			
	Mean	Min	Max	Std	Mean	Min	Max	Std
Data Selection and Preprocessing	36.1	26.9	68.0	7.0	29.6	18.8	48.0	5.0
Registration	85.7	53.8	197.4	17.4	76.7	49.1	168.8	15.4
Lesion Segmentation	5.7	4.9	10.8	1.0	4.1	3.9	4.6	0.2
Outcome Prediction	1.0	0.9	1.1	0.0	0.9	0.8	0.9	0.0
Report Generation	146.3	101.6	195.2	21.6	10.0	7.8	15.8	1.65
**Total**	**274.8**	**188.1**	**472.5**	**47.0**	**121.3**	**80.4**	**238.1**	**22.3**

References

1. M. R. Hernandez Petzsche, E. de la Rosa, U. Hanning, R. Wiest, W. Valenzuela, M. Reyes, M. Meyer, S.-L. Liew, F. Kofler, I. Ezhov, D. Robben, A. Hutton, T. Friedrich, T. Zarth, J. Bürkle, T. A. Baran, B. Menze, G. Broocks, L. Meyer, C. Zimmer, T. Boeckh-Behrens, M. Berndt, B. Ikenberg, B. Wiestler, J. S. Kirschke, ISLES 2022: A multi-center magnetic resonance imaging stroke lesion segmentation dataset. *Sci. Data* **9**, 762 (2022).

2. G. H. Mc Laughlin, SMOG Grading-a New Readability Formula. *J. Read.* **12**, 639–646 (1969).

3. M. Kutner, E. Greenberg, Y. Jin, C. Paulsen, *The Health Literacy of America's Adults: Results From the 2003 National Assessment of Adult Literacy (NCES 2006–483)* (Washington, DC, 2006).

Personalized Stroke Report

Overview

Study Information

Date: [redacted]   Study ID: [redacted]   [removed to preserve confidentiality]

Stroke Anatomy

The system identified an ischemic stroke lesion with a total lesion volume of approximately 74.7 cm^3, about the size of 2 golf_balls. The lesion is located in the left hemisphere, primarily within the arterial distribution of the middle cerebral artery. Additional details of the lesion's anatomy are provided below:

- **Hemispheric and White/Gray Matter**: 100.0% of the lesion is in the left hemisphere, with 3.8% of the lesion in white matter and 96.3% in gray matter.
- **Anatomical**: The lesion is primarily in the frontal lobe affecting the middle frontal gyrus and precentral gyrus.
- **Arterial**: The lesion is primarily in the region of the middle cerebral artery.
- **White Matter Tracts**: The lesion primarily affects the superior longitudinal fasciculus 2, frontal aslant tract, and corticostriatal tract superior.

The visualized lesion on clinical MRI and 3D lesion reconstructions is presented on Page 2, with subsequent pages containing tables of detailed neuroanatomical analysis.

Outcome Predictions

- **Expressive Language**: High Risk
- **Receptive Language**: High Risk
- **Visuospatial Ability**: High Risk
- **Working Memory**: High Risk
- **Executive Function**: High Risk

Risk Assessment

The location and characteristics of the stroke have been associated with a higher risk of Expressive Language, Receptive Language, Visuospatial Ability, Working Memory, and Executive Function impairments. This does not mean you will definitely experience these problems, only that many people may have difficulties in these areas after a stroke.

Recommendations

- **Cognitive Rehabilitation:** Given the predicted high risk for cognitive impairments, a referral for evaluation and dedicated cognitive rehabilitation may be beneficial.
- **Neuropsychology Referral:** Given the predicted high risk for impairments in working memory, executive functions, visuospatial abilities, a referral to a neuropsychologist for detailed assessment may be beneficial.
- **Speech Therapy:** Given the predicted high risk for impairments in expressive language, receptive language, a referral to a speech therapist for assessment may be beneficial.
- **Early Rehabilitation:** For patients identified as high risk for cognitive impairments, initiating rehabilitation within the first 3 months post-stroke is recommended to capitalize on peak neuroplasticity. Intensive focus during the first 6 months may significantly enhance recovery potential, as research indicates limited gains in the chronic phase thereafter.
- **Monitoring:** Regular follow-ups to monitor the progression of impairments and adjust treatment plans accordingly.

This assessment is not meant to be comprehensive and evaluates risks for select cognitive functions post-stroke. This report is meant to support clinical judgment, not replace it. It requires a full clinical evaluation and ongoing monitoring due to individual variability. Continuous, tailored care is key to recovery.

NOT FOR CLINICAL USE • NOT FOR CLINICAL USE
NOT FOR CLINICAL USE • NOT FOR CLINICAL USE

The University of Iowa © 2025

NOT FOR CLINICAL USE • NOT FOR CLINICAL USE

Radiology

Radiology Images:

Trace-weighted Image

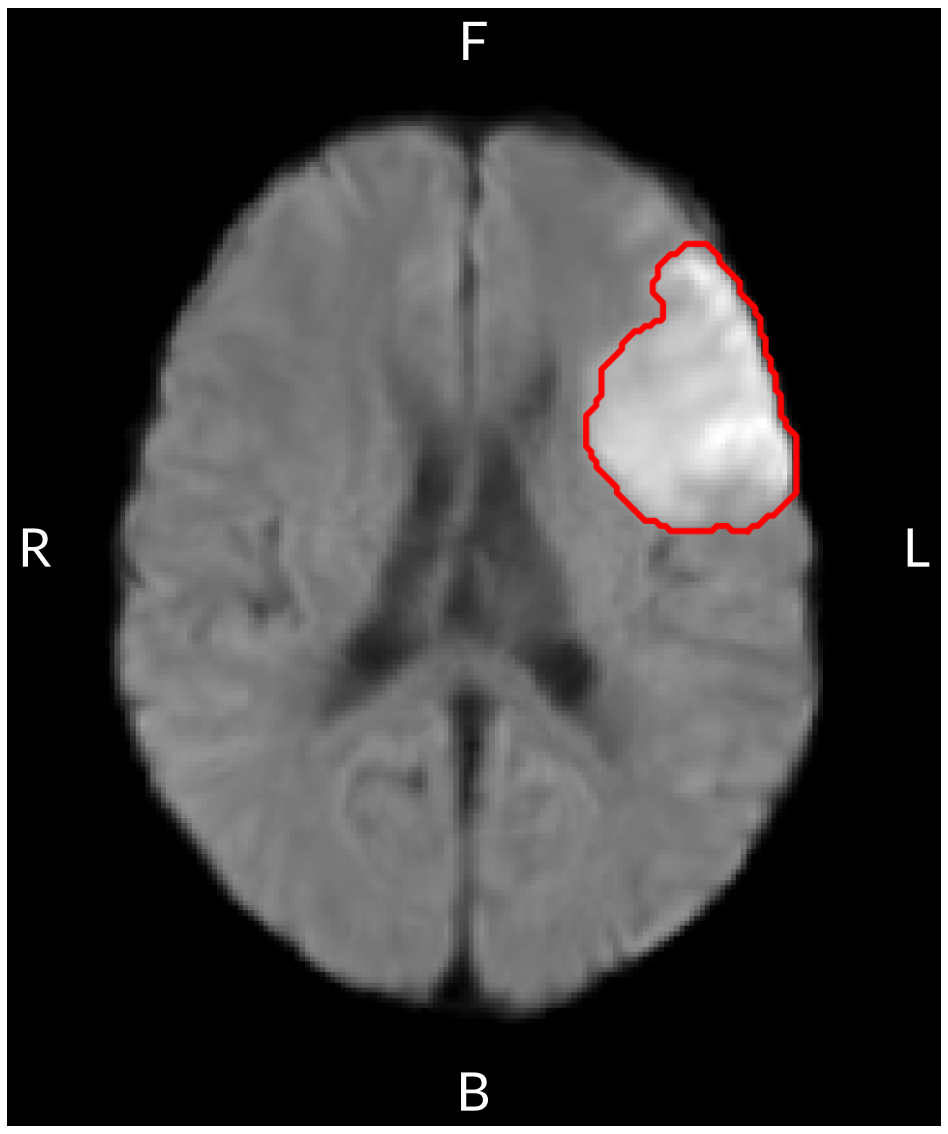

ADC Image

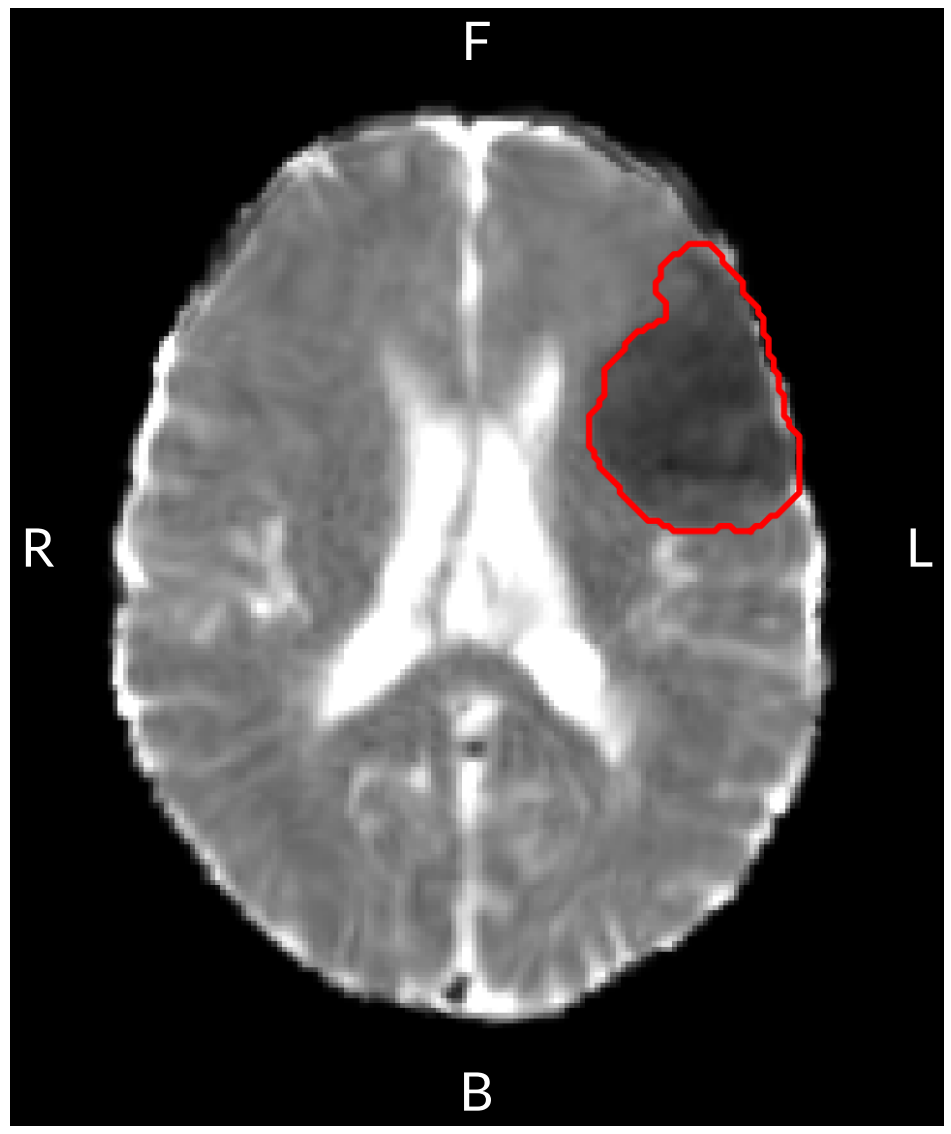

Anatomical Image

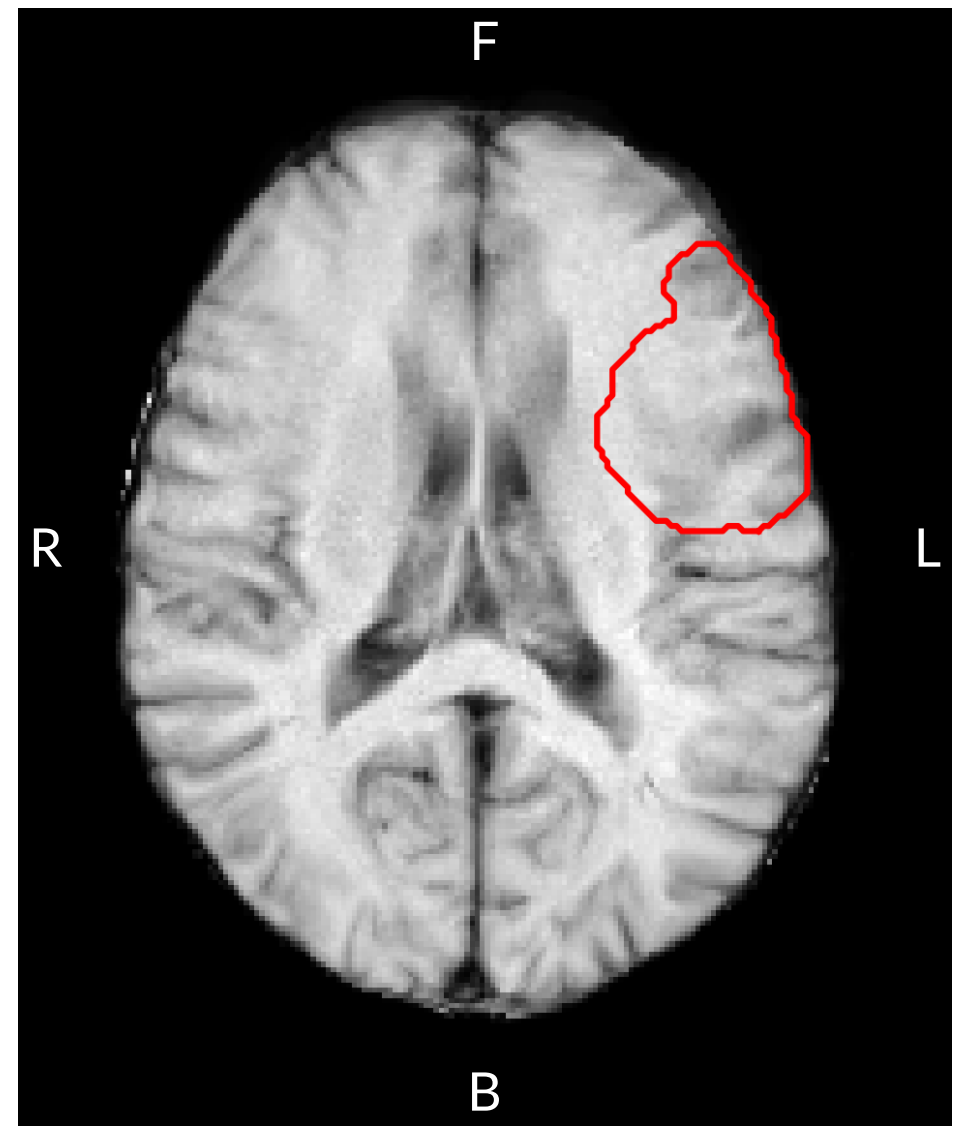

3D Renderings:

Axial View

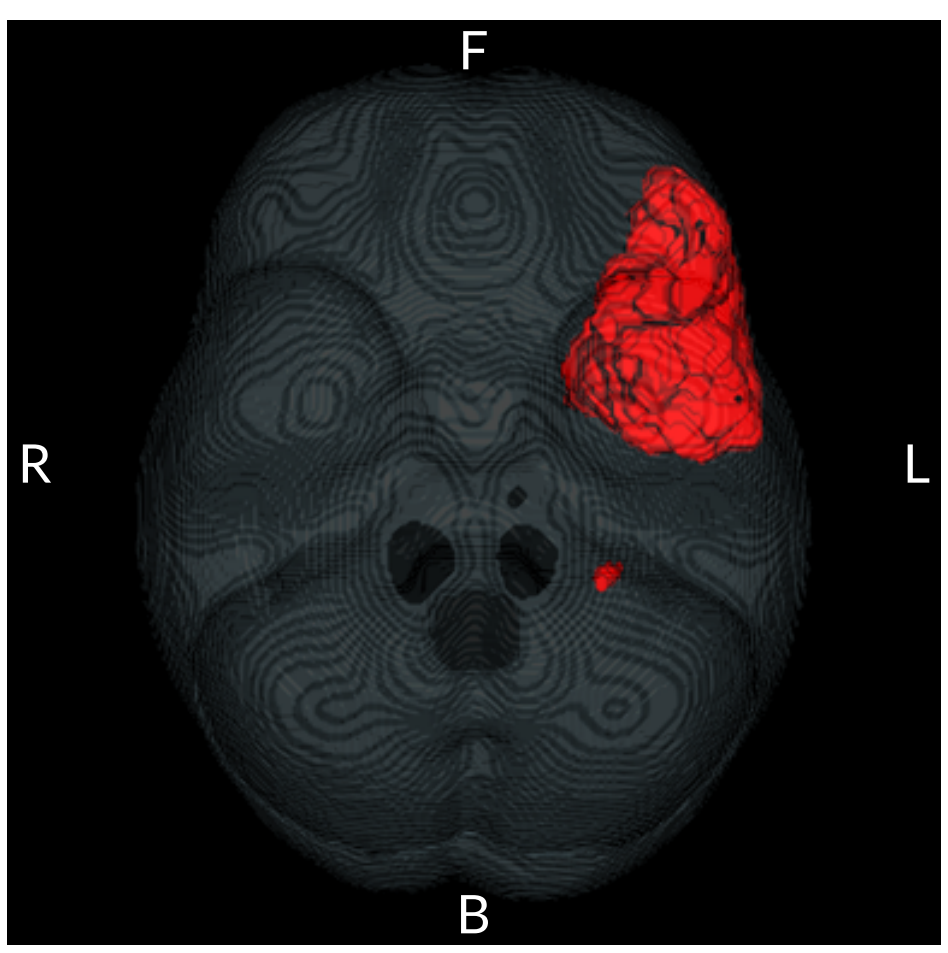

Coronal View

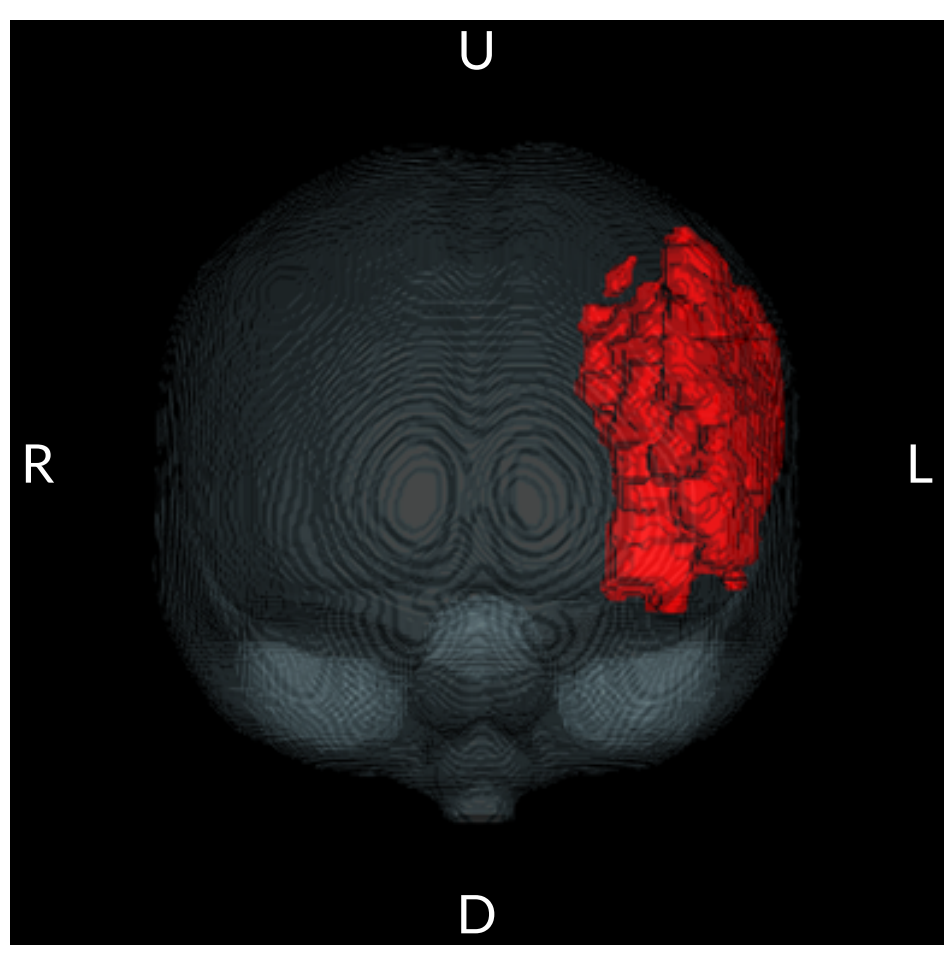

Sagittal View

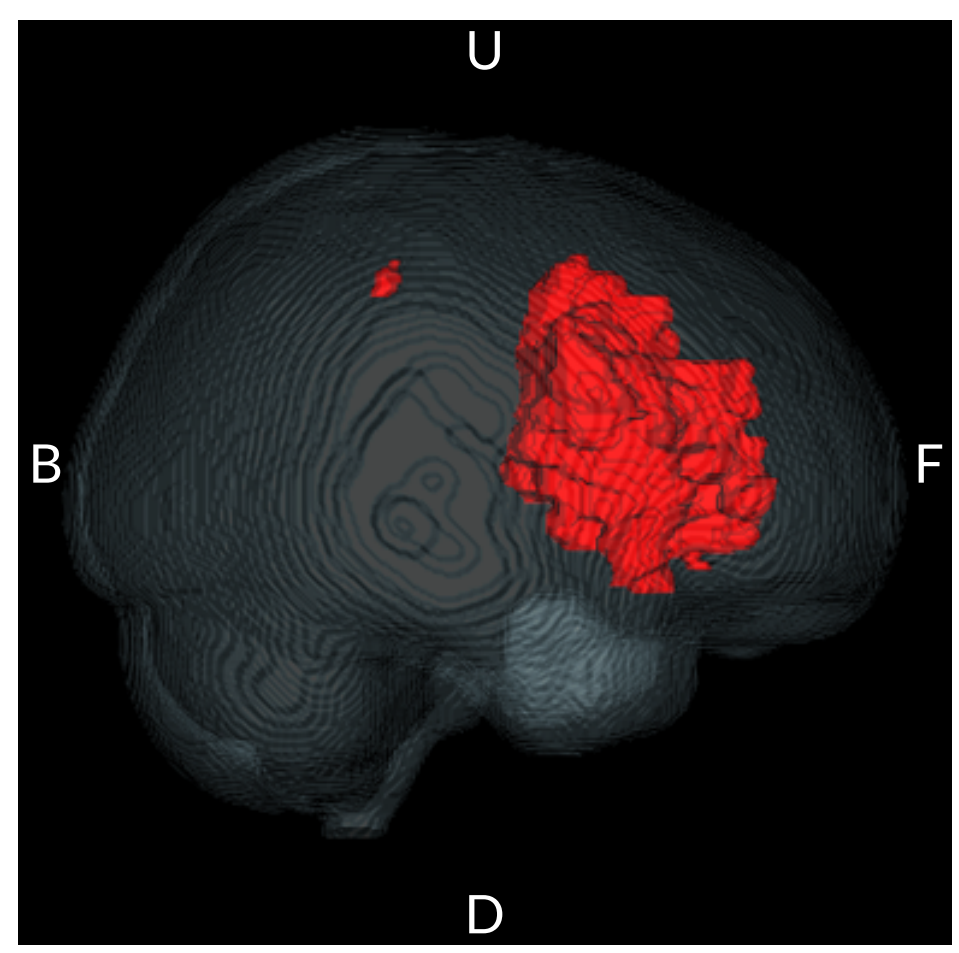

Note: The radiological images (DWI, ADC, Anatomical) are presented in radiological orientation, where the right side of the image corresponds to the patient's left hemisphere and vice versa; orientation labels (**L**: Left, **R**: Right, **U**: Up, **D**: Down, **F**: Front, **B**: Back) are included for clarity. The 3D renderings below visualize the lesion volume in axial, coronal, and sagittal views, using the same orientation labels.

NOT FOR CLINICAL USE • NOT FOR CLINICAL USE

The University of Iowa © 2025

IOWA

NOT FOR CLINICAL USE • NOT FOR CLINICAL USE

Anatomical Structure Analysis

Hemisphere	Region	Subregion	Lesion Volume (cm^3)	[%] of Lesion	[%] of Region
Left Hemisphere	Frontal Lobe	Middle Frontal Gyrus	14.50	19.5	41.8
		Precentral Gyrus	13.80	18.5	25.3
		Inferior Frontal Gyrus (Pars Opercularis)	8.10	10.8	84.0
		Inferior Frontal Gyrus (Pars Triangularis)	7.20	9.6	75.9
		Frontal Pole	6.50	8.7	7.2
		Central Opercular Cortex	5.30	7.2	52.0
		Frontal Operculum	4.00	5.3	96.3
		Frontal Orbital Cortex	3.90	5.2	18.6
	Insular Cortex	Insular Cortex	7.50	10.0	47.0
	White Matter	Cerebral WM	2.80	3.8	4.1
	Parietal Lobe	Postcentral Gyrus	0.50	0.7	1.1
		Superior Parietal Lobule	0.20	0.2	0.9
	Temporal Lobe	Planum Polare	0.30	0.4	6.5
		Temporal Pole	0.10	0.2	0.4

NOT FOR CLINICAL USE • NOT FOR CLINICAL USE

The University of Iowa © 2025

NOT FOR CLINICAL USE • NOT FOR CLINICAL USE

Lesion White and Gray Matter Distribution

Matter Type	[%] of Lesion
White Matter	3.75%
Gray Matter	96.25%

Arterial Structure Analysis

Hemisphere	Region	Subregion	Lesion Volume (cm^3)	[%] of Lesion	[%] of Region
Left Hemisphere	Middle Cerbral Artery	Frontal Pars Of Middle Cerebral Artery	65.70	88.0	40.8
		Insular Pars Of Middle Cerebral Artery	5.70	7.6	44.3
		Temporal Pars Of Middle Cerebral Artery	1.00	1.4	0.8
		Parietal Pars Of Middle Cerebral Artery	0.90	1.3	0.9
		Lateral Lenticulostriate	0.80	1.0	3.0
	Anterior Cerebral Artery	Anterior Cerebral Artery	0.20	0.3	0.1

The University of Iowa © 2025

NOT FOR CLINICAL USE • NOT FOR CLINICAL USE

White Matter Structure Analysis

Hemisphere	Type	Subregion	Lesion Volume (cm^3)	[%] of Lesion	[%] of Region
Left Hemisphere	Association	Superior Longitudinal Fasciculus 2	10.80	14.5	29.2
		Frontal Aslant Tract	8.90	11.9	57.4
		Superior Longitudinal Fasciculus 3	7.20	9.7	46.8
		Arcuate Fasciculus	6.50	8.7	42.9
		Inferior Fronto-Occipital Fasciculus	2.30	3.1	6.8
		Extreme Capsule	0.70	1.0	12.7
		Uncinate Fasciculus	0.20	0.3	2.7
	Projection	Corticostriatal Tract Superior	7.60	10.2	22.6
		Thalamic Radiation Superior	7.40	9.9	20.2
		Corticopontine Tract Frontal	5.30	7.1	15.2
		Corticobulbar Tract	2.90	3.9	47.7
		Corticostriatal Tract Anterior	1.80	2.4	8.6
		Reticulospinal Tract	1.70	2.3	14.6
		Dentatorubrothalamic Tract	1.50	2.0	8.8
		Corticospinal Tract	1.20	1.6	7.1
		Thalamic Radiation Anterior	0.80	1.1	5.7
		Corticopontine Tract Parietal	0.20	0.2	0.6
Other	Commissural	Corpus Callosum Mid Anterior	6.30	8.4	15.4
		Corpus Callosum Central	4.80	6.5	10.4
		Corpus Callosum Anterior	3.60	4.8	5.7
		Corpus Callosum Mid Posterior	0.40	0.6	0.9
		Corpus Callosum Posterior	0.10	0.1	0.1

NOT FOR CLINICAL USE • NOT FOR CLINICAL USE

The University of Iowa © 2025

Outcome Prediction

	Unable to Assess	Low Risk	High Risk
**Expressive Language**	☐	☐	X
**Receptive Language**	☐	☐	X
**Visuospatial Ability**	☐	☐	X
**Working Memory**	☐	☐	X
**Executive Function**	☐	☐	X

Expressive language refers to the ability to translate one's thoughts and feelings to others. This includes the ability to speak and write. People with deficits in this domain may have trouble producing the names of items or communicating in full sentences.

Receptive language refers to the ability to comprehend language-related information. A common way to assess receptive language is to give someone instructions to follow and see if they are able to make sense of the instructions and produce the requested behavior. For example, if you were told to use your left thumb to touch your right ear and then did that it would suggest you understood the request and you were able to carry it out.

Visuospatial ability refers to your ability to solve tasks that are typically visual in nature and may require the mental or physical manipulation of objects on paper or in space.

Working Memory refers to the ability to temporarily hold and manipulate information in mind. This cognitive function is essential for many daily activities such as following conversations or instructions. For example, if you were given a phone number and told to remember it for 30 seconds before dialing, this would require working memory. Similarly, mental calculations, remembering driving directions while navigating, or keeping track of items while shopping all rely on this ability.

Executive Function refers to higher-level cognitive processes that enable planning, flexible thinking, self-regulation, and goal-directed behavior. This includes skills such as organizing information, switching between tasks, inhibiting inappropriate responses, and monitoring one's own performance. Our prediction is primarily based on performance on the Trail Making Test, which requires connecting alternating sequences (1-A-2-B-3-C, etc.), assessing cognitive flexibility and the ability to coordinate multiple cognitive processes simultaneously.

NOT FOR CLINICAL USE • NOT FOR CLINICAL USE
NOT FOR CLINICAL USE • NOT FOR CLINICAL USE

The University of Iowa © 2025

NOT FOR CLINICAL USE • NOT FOR CLINICAL USE

Patient Section

What Happened

You've had an ischemic stroke. This is when blood flow to part of your brain gets blocked. This blockage stops oxygen delivery to brain cells. Without oxygen, these cells can become damaged.

Let's look at the pictures on Page 2. You'll see images of your brain. The red line shows where the damage is. The damaged area is about the size of 2 golf_balls. This damage is primarily in the region responsible for motor and cognitive function.

This is not your fault. According to the American Heart Association, over 800,000 people in the United States experience a stroke each year. You are not alone in this struggle.

What to Expect

The road to recovery is a journey that requires patience, dedication, and support. Here's what you can expect during the different phases of your rehabilitation:

Initial Stabilization (0-2 weeks)

Focus on regaining basic functions such as walking, speaking, and swallowing. This phase is a foundation for further recovery. The goal is to help you become stable to safely return home.

First 3 months - Critical Recovery Period

This is the most important time for your recovery. Your brain has the greatest ability to heal during these months. Early and consistent rehabilitation leads to better outcomes. Work closely with your therapists during this time. Your rehabilitation team will develop a personalized plan tailored to your needs.

First year progress

Most recovery happens during the first six months. After that, progress will slow down but continue. You may see rapid improvements at first, then more gradual changes. Some challenges may remain. Continue working with your healthcare team to adjust your treatment plan as needed.

Long-term outlook

Recovery continues as a lifelong process. You will learn strategies to work around any remaining difficulties. Many people find new ways to accomplish daily tasks. With support and practice, you can build a fulfilling life after stroke. Focus on what you can do, rather than what you can't.

NOT FOR CLINICAL USE • NOT FOR CLINICAL USE

The University of Iowa © 2025

Personalized Stroke Report

IOWA

NOT FOR CLINICAL USE • NOT FOR CLINICAL USE

Your Risks

Based on the stroke location in your brain we can try to predict what types of functions could be disrupted. However, it is important to know each stroke is different and there is no way to know with certainty what types of problems you may experience.

Expressive Language: You are at **higher risk** for long-term difficulty with speaking or writing. You may find it hard to find words or form sentences. Speech therapy and communication strategies will be important tools in your recovery.

Receptive Language: You are at **higher risk** and face lasting challenges understanding spoken or written language. Following conversations or reading may be difficult.

Visuospatial Ability: You are at **higher risk** and may have lasting trouble with visual tasks like judging space, recognizing objects, or following directions.

Working Memory: You are at **higher risk** and may have difficulty holding and using short-term information. This can affect daily tasks like remembering instructions.

Executive Function: You are at **higher risk** and may face long-term challenges with planning, decision-making, or staying organized. Support through therapy and structured routines can help you manage these difficulties.

What to Do

Watch For These Important Signs:

- If you notice problems with movement, speech, thinking, or daily tasks, you may need more help. Talk to your doctor about any issues.
- Be alert for warning signs of another stroke: sudden numbness, confusion, trouble seeing, bad headache, or trouble walking. If you notice these, get medical help right away.

To ensure a successful recovery:

- Early therapy is very important during the first three months. Contact your doctor and therapy specialists as soon as possible to set up your plan.
- Regular therapy will help you recover and keep making progress.
- Family support is crucial for your recovery. Ask them to be involved throughout the process.

Specific actions include:

- Following your therapy plan
- Eating healthy foods
- Moving regularly, such as walking or doing exercises your therapist shows you
- Practicing new skills with help from family members
- Watching for signs of stroke as mentioned above

Remember that recovery is a journey, not a destination. With hard work, patience, and support, you will overcome this challenge.

NOT FOR CLINICAL USE • NOT FOR CLINICAL USE

The University of Iowa © 2025
